# Metal-defect pairs for near-stoichiometric electrocatalytic C–N coupling

**DOI:** 10.1093/nsr/nwag206

**Published:** 2026-04-09

**Authors:** Yandong Wu, Wei Chen, Yuqin Zou, Jinbo Wang, Ruiqi Wang, Nan Hu, Qie Liu, Mengyi Qiu, Yimin Jiang, Mengwei Han, Ming Yang, Leitao Xu, Jiawang Chen, Chao Xie, Dongdong Wang, Yabin Xu, Yongmin He, Xuning Li, Liwu Fan, Xiang Gao, Shuangyin Wang

**Affiliations:** State Key Laboratory of Chem/Bio-Sensing and Chemometrics, College of Chemistry and Chemical Engineering, Hunan University, Changsha 410082, China; State Key Laboratory of Chem/Bio-Sensing and Chemometrics, College of Chemistry and Chemical Engineering, Hunan University, Changsha 410082, China; State Key Laboratory of Chem/Bio-Sensing and Chemometrics, College of Chemistry and Chemical Engineering, Hunan University, Changsha 410082, China; State Key Laboratory of Chem/Bio-Sensing and Chemometrics, College of Chemistry and Chemical Engineering, Hunan University, Changsha 410082, China; State Key Laboratory of Chem/Bio-Sensing and Chemometrics, College of Chemistry and Chemical Engineering, Hunan University, Changsha 410082, China; Department of Mechanical and Aerospace Engineering, Princeton University, Princeton, NJ 08544, USA; State Key Laboratory of Clean Energy Utilization, Zhejiang University, Hangzhou 310027, China; State Key Laboratory of Chem/Bio-Sensing and Chemometrics, College of Chemistry and Chemical Engineering, Hunan University, Changsha 410082, China; State Key Laboratory of Chem/Bio-Sensing and Chemometrics, College of Chemistry and Chemical Engineering, Hunan University, Changsha 410082, China; State Key Laboratory of Chem/Bio-Sensing and Chemometrics, College of Chemistry and Chemical Engineering, Hunan University, Changsha 410082, China; State Key Laboratory of Chem/Bio-Sensing and Chemometrics, College of Chemistry and Chemical Engineering, Hunan University, Changsha 410082, China; State Key Laboratory of Chem/Bio-Sensing and Chemometrics, College of Chemistry and Chemical Engineering, Hunan University, Changsha 410082, China; State Key Laboratory of Chem/Bio-Sensing and Chemometrics, College of Chemistry and Chemical Engineering, Hunan University, Changsha 410082, China; State Key Laboratory of Chem/Bio-Sensing and Chemometrics, College of Chemistry and Chemical Engineering, Hunan University, Changsha 410082, China; State Key Laboratory of Chem/Bio-Sensing and Chemometrics, College of Chemistry and Chemical Engineering, Hunan University, Changsha 410082, China; College of Chemistry and Chemical Engineering, Institute of Interdisciplinary Studies, Hunan Normal University, Changsha 410081, China; State Key Laboratory of Chem/Bio-Sensing and Chemometrics, College of Chemistry and Chemical Engineering, Hunan University, Changsha 410082, China; State Key Laboratory of Chem/Bio-Sensing and Chemometrics, College of Chemistry and Chemical Engineering, Hunan University, Changsha 410082, China; State Key Laboratory of Chem/Bio-Sensing and Chemometrics, College of Chemistry and Chemical Engineering, Hunan University, Changsha 410082, China; State Key Laboratory of Catalysis, Dalian Institute of Chemical Physics, Chinese Academy of Sciences, Dalian 116023, China; State Key Laboratory of Clean Energy Utilization, Zhejiang University, Hangzhou 310027, China; State Key Laboratory of Clean Energy Utilization, Zhejiang University, Hangzhou 310027, China; State Key Laboratory of Chem/Bio-Sensing and Chemometrics, College of Chemistry and Chemical Engineering, Hunan University, Changsha 410082, China

**Keywords:** near-stoichiometric conversion, C–N coupling, electrocatalysis, metal-defect pairs

## Abstract

Geminal-site catalysts (GSCs) are prospective candidates for fulfilling the goal of aqueous electrochemical reductive cross-coupling reactions (ERCR) at near-stoichiometric yields. Nevertheless, a problem lies in the lack of a synthetic route for GSCs with few single sites. Here we report a defect-accompanying strategy for synthesizing GSCs containing metal-defect catalytic pairs (M-D GSCs), meaning that Fe-D GSCs can realize the electrochemical synthesis of cyclohexanone oximes (CHOs) from high concentrations (0.5 M) of nitrites (NO_2_^−^) and cyclohexanone (CYC) at near-stoichiometric yields (the Faradic efficiency or yield_C/N_: 91.3%). Multiple *in-*/*ex-situ* characterizations demonstrated that metal-citrate complexes were converted to metal-defect catalytic pairs via the liberation of gaseous carbon/nitrogen species during pyrolysis. Furthermore, we developed an innovative cathodic oxime-alkali process, where high concentration NaNO_2_ and CYC can be electrochemically converted to high-purity products including NaOH and CHO. This work showcases the enormous potential of M-D GSCs in achieving near-stoichiometric conversion for ERCR reactions.

## Teaser

This work firstly reports the near stoichiometric synthesis of cyclohexanone oxime (the essential precursor for nylon-6) on Fe-defect geminal-site catalysts with high faradaic efficiency

## INTRODUCTION

**Figure 1.**
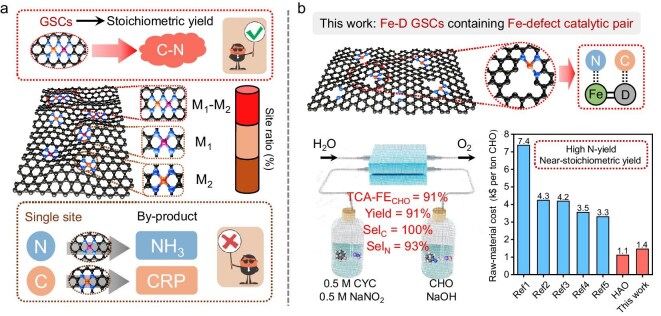
Schematic of near-stoichiometric conversion of electrocatalytic CHO synthesis on GSCs. (a) Challenges for the near-stoichiometric conversion of electrocatalytic C–N coupling on GSCs. (b) Depicting the electrocatalytic synthesis of CHO from NaNO_2_ and CYC at near-stoichiometric yields on Fe-D GSCs synthesized via an accompanying defect strategy. The illustration compares the raw-material cost of per ton CHO of this work, mature ammoximation process (HAO), and previous reports [12–16].

Ideal geminal-site catalysts (GSCs) without single sites are expected to achieve stoichiometric yields of aqueous electrocatalytic reductive cross-coupling reactions (ERCRs), e.g. the electrocatalytic synthesis of cyclohexanone oximes (CHO) from cyclohexanones (CYC) and nitrites (NO_2_^−^) [1–4]. Commonly, heterogeneous geminal sites can catalyze the formation of cross-coupling products, while single catalytic sites favor the conversion of single molecules into by-products (Fig. 1a) [5–8]. It is essential to eliminate single catalytic sites in GSCs for the target of near-stoichiometric conversion for ERCR. However, the formation of single sites is usually more thermodynamically favorable than that of geminal catalytic sites. Consequently, it is extremely difficult to synthesize ideal GSCs without single sites [9–11].

Take carbon-based catalysts for instance, defects can activate adjacent inert sites so that carbon defects usually exhibit excellent catalytic characteristics in electrocatalytic reactions [17–20]. Hence, based on carbon-based single-metal-site catalysts, single metal sites can be transformed into the geminal sites of metal-defect catalytic pairs via the introduction of accompanying defects. For example, theoretically, the pyrolysis deoxidation of metal-carboxylate complexes can lead to the formation of metal-defect (M-D) catalytic pairs, and this transformation is thermodynamically favored. Consequently, this is a promising and alternative approach to synthesize ideal GSCs with few single sites. But even if ideal GSCs can be synthesized, the following tough challenges still hinder the stoichiometric yields for ERCR: (1) how to ensure the co-adsorption and cross-coupling of key intermediates on each metal-defect catalytic pair; (2) how to eliminate side reactions, e.g. the over-reduction of key intermediates.

Herein, we propose a novel synthesis method of geminal-site catalysts containing metal-defect catalytic pairs (M-D GSCs) via pyrolyzing metal-citric acid complexes loaded on a carbon support. Therefore, Fe-D GSCs can catalyze the electrocatalytic synthesis of CHO from CYC and NO_2_^−^ in a near-stoichiometric manner, with the yield_N/C_ and Faradic efficiency of CHO being as high as 91.3% at theoretical transfer coulomb amounts in order to obtain complete conversion (Fig. 1b). Multiple *in-*/*ex-situ* characterizations prove that ferrous-citrate complexes can be converted to Fe-defect catalytic pairs (Fe-D CP) on a carbon support during pyrolysis via the liberation of gaseous carbon/nitrogen species (e.g. CO and NO). Owing to the thermodynamic advantage for the formation of Fe-D CP, almost every isolated Fe site borders on one nitrogen defect and several carbon defects in Fe-D GSCs. Through a combination of experiments and simulations, Fe-D CP can not only realize the selective reduction of NO_2_^−^ to adsorbed hydroxylamine (*NH_2_OH) but also enable almost all *NH_2_OH to C–N couple with adsorbed CYC (*CYC) to generate CHO. Moreover, we develop an innovative cathodic oxime-alkali process under near-stoichiometric feeding at high concentrations, where 0.55 M NaNO_2_ and 0.5 M CYC could be converted to highly pure CHO (0.17 g CHO h^−1^) and NaOH solution via the flow reactor system.

## RESULTS AND DISCUSSION

### Screening isolated metal sites for the selective reduction of NO_2_^−^ to *NH_2_OH

CHO synthesis is one of the most important processes in the nylon industry, and to meet the growing demand, global production will need to increase to 8.9 million metric tons per annum [21]. Electrocatalytic synthesis of CHO from CYC and NO_2_^−^ is a promising alternative strategy for CHO synthesis due to the advantages of being energy-saving and environmentally friendly so that it is chosen here as the model reaction of ERCR [12,13,22,23]. The most optimal solution for the industrialization of ERCR is to achieve stoichiometric conversion and yield based on ideal catalysts [24,25]. Although previous studies reported some catalysts for the electrocatalytic synthesis of CHO, the performances of these catalysts cannot fulfil the target of near-stoichiometric yields [12,13,22,23]. We carried out stoichiometry feeding at high concentrations (0.5 M NO_2_^−^ and 0.5 M CYC) in this work, and most performance parameters were recorded at near-complete conversion. In order to accurately evaluate the industrial prospects of catalysts, this work focused on the Faradic efficiency of CHO at the theoretical transfer coulomb amount for complete conversion (TCA-FE_CHO_), and found that the yield_C/N_ of CHO is the same as the TCA-FE_CHO_ in this system.

Electrocatalytic synthesis of CHO from NO_2_^−^ and CYC consists of the following three processes: (1) selective electrocatalytic reduction of NO_2_^−^ to *NH_2_OH; (2) adsorption of CYC adjacent to existing *NH_2_OH; and (3) C–N coupling of *NH_2_OH and *CYC towards CHO (Fig. S1). More importantly, CHO selectivity depends on the effective collision frequency between *NH_2_OH and *CYC, which is highly relevant to the molecule spacing. Due to the uncertainty of CYC adsorption, only parts of *NH_2_OH can capture adjacent *CYC for C–N coupling, while other *NH_2_OH intermediates favor over-reduction in order to form by-products, e.g. ammonia (Fig. S2). As a result, traditional catalysts seem impossible to realize the near-stoichiometric conversion towards CHO. If geminal-site catalytic pairs can enable each *NH_2_OH to react with adjacent *CYC to generate CHO, ideal GSCs with few single sites are expected to realize the near-stoichiometric conversion towards CHO. Metal sites are considered to be active sites for an electrocatalytic nitrite reduction reaction (NO_2_RR) [26–30]. On the other hand, defect sites, especially carbon defects, usually show the specific adsorption of carbonyl compounds, such as CYC [31–34]. Therefore, M-D GSCs containing metal-defect catalytic pairs have a great potential for near-stoichiometric conversion towards CHO.

**Figure 2.**
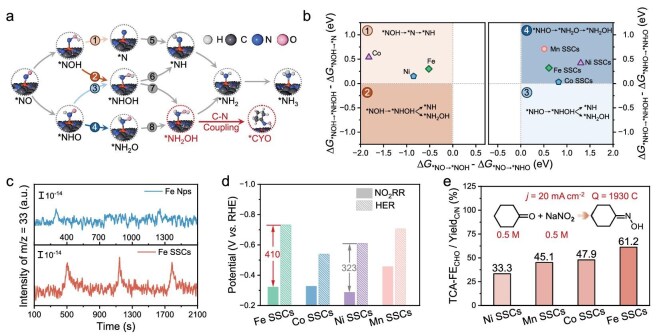
Selective reduction of NO_2_^−^ to *NH_2_OH on different isolated metal sites. (a) Schematic diagrams of possible NO_2_RR pathways. (b) Comparison of free energy changes for the hydrogenation of *NOH (left) and *NHO (right) vs free energy changes for the hydrogenation of *NO as an indicator for identifying the NO_2_RR pathways of metal catalysts and metal SSCs. (c) Real-time profiles corresponding to the generation of *NH_2_OH (33 *m*/*z*) during the NO_2_RR over Fe Nps and Fe SSCs in the DEMS measurement. (d) Differences of reaction potentials between NO_2_RR and HER over M SSCs (Fe, Co, Ni, and Mn). (e) TCA-FE_CHO_ and yield_C/N_ of M SSCs (M: Fe, Co, Ni, and Mn) for electrochemical CHO synthesis from 0.5 M NaNO_2_ and 0.5 M CYC (electrolyte: 0.5 M Na_2_CO_3_) at theoretical coulomb amounts (1930 C).

Given that *NH_2_OH is the key intermediate for C–N coupling, the NO_2_RR pathway over metal sites must follow the path involving *NH_2_OH for stoichiometric conversion towards CHO on M-D GSCs (Fig. 2a and Fig. S3) [35–37]. According to density functional theory (DFT) calculations, bulk metals (i.e. Co, Ni, and Fe) are unsuitable for near-stoichiometric conversion towards CHO because NO_2_RR pathways on these bulk metals follow the pathway involving *NOH→*N→*NH (Pathway 1→5), instead of the pathway involving *NH_2_OH (Figs S4 and S5, and Table S1). As predicted, both TCA-FE_CHO_ and yield_C/N_ are all below 25% for electrocatalytic CHO synthesis on Fe, Co , and Ni metal nanoparticles, which are much lower compared with the goal of ‘the stoichiometric yield’ (Figs S6–S8). Single metal sites (e.g. Fe, Co, Ni, and Mn), in theory, mainly follow the pathway involving *NHO→*NH_2_O→*NH_2_OH (Pathway 4→8) (Fig. 2b, Figs S9 and S10, and Table S2). Accordingly, a series of single-metal-site catalysts (M SSCs; M: Fe, Co, Ni, and Mn) were synthesized and characterized (Figs S11–S13). Differential electrochemical mass spectrometry (DEMS) results further confirm that Fe SSCs favor the enrichment of *NH_2_OH during NO_2_RR (Fig. 2c and Fig. S14).

In order to maximize the TCA-FE_CHO_ of CHO, the ideal catalysts have not only an excellent NO_2_RR performance for *NH_2_OH formation but also a poor activity of competition reactions, e.g. hydrogen evolution reaction (HER). We screened out suitable isolated metal sites through comparing the differences of reaction potentials between NO_2_RR and HER based on different M SSCs. Fe SSCs have the largest difference (410 mV) of reaction potentials between NO_2_RR and HER (Fig. 2d and Fig. S15). Accordingly, both TCA-FE_CHO_ and yield_C/N_ on Fe SSCs (61.2%) are highest for electrocatalytic CHO synthesis among a series of M SSCs (Fig. 2d and Fig. S16). Hence, we speculate that the isolated metal site in metal-defect catalytic pairs favor the electroreduction of NO_2_^−^ to *NH_2_OH, instead of HER. Therefore, Fe-D GSCs containing Fe-defect catalytic pairs may be an ideal catalyst for the near-stoichiometric conversion towards CHO at the TCA-FE_CHO_.

### Impact of Fe-defect catalytic pairs on CYC adsorption and C–N coupling

**Figure 3.**
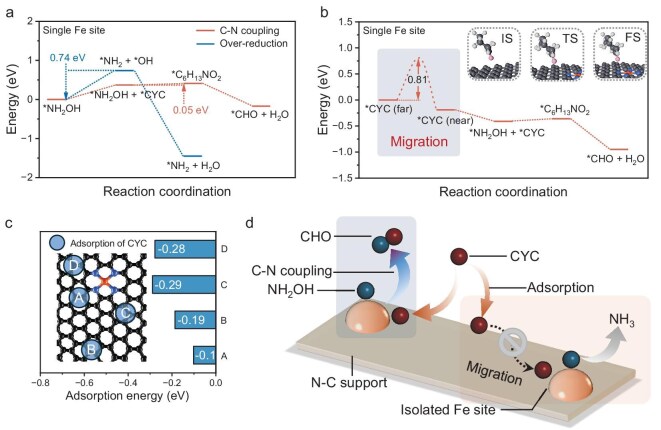
CYC adsorption on C–N coupling over the single Fe site. (a) Energy profiles of C–N coupling and *NH_2_OH reduction on the Fe SSC model catalyst adsorbed with *CYC and *NH_2_OH (*CYC is close to *NH_2_OH). (b) Energy profiles of *CYC migration and C–N coupling on the Fe SSC model catalyst adsorbed with *CYC and *NH_2_OH (*CYC is far away from *NH_2_OH). (c) Adsorption energy of CYC at different sites on the Fe SSC model catalyst. (d) Schematic diagram showing the evolution of *NH_2_OH and *CYC on the Fe SSC.

Another constraint on the TCA-FE_CHO_ and yield_C/N_ for CHO formation is the uncertainty of CYC adsorption. Accordingly, we investigate the influence of the distance between *CYC and *NH_2_OH on the C–N coupling. When *CYC is close to *NH_2_OH, the free energy change of C–N coupling (0.05 eV) is much lower than that of *NH_2_OH reduction (0.74 eV), which proves that the co-adsorption of *NH_2_OH and *CYC favors C–N coupling towards CHO, instead of the electroreduction of *NH_2_OH (Fig. 3a, Fig. S17, and Table S3). When *CYC is far away from *NH_2_OH, *CYC must move to the site adjacent to *NH_2_OH for C–N coupling to occur. However, the migration of *CYC needs to overcome the high energy barrier of 0.81 eV, thus resulting in the limitation of subsequent C–N coupling (Fig. 3b, Fig. S18, and Table S4).

For the structure of Fe SSCs, CYC adsorption energy of the site close to the single Fe site (e.g. A site: −0.1 eV) is higher than those for distant sites (e.g. B site: −0.19 eV; C site: −0.29 eV; D site: −0.28 eV), which proves that the single Fe site can not specifically adsorb CYC (Fig. 3c, Fig. S19, and Table S5). Due to a lack of specific adsorption of CYC adjacent to *NH_2_OH for Fe SSCs, some *NH_2_OH intermediates can be electrocatalytically reduced to the by-product of NH_3_. This is why the Faradic efficiency of NH_3_ for Fe SSCs still exceeds 30% for electrocatalytic CHO synthesis on Fe SSCs (Fig. S16). The key for near-stoichiometric conversion towards CHO is to prevent the over-reduction of *NH_2_OH, and the best bet would be to ensure the co-adsorption between *NH_2_OH and *CYC.

**Figure 4.**
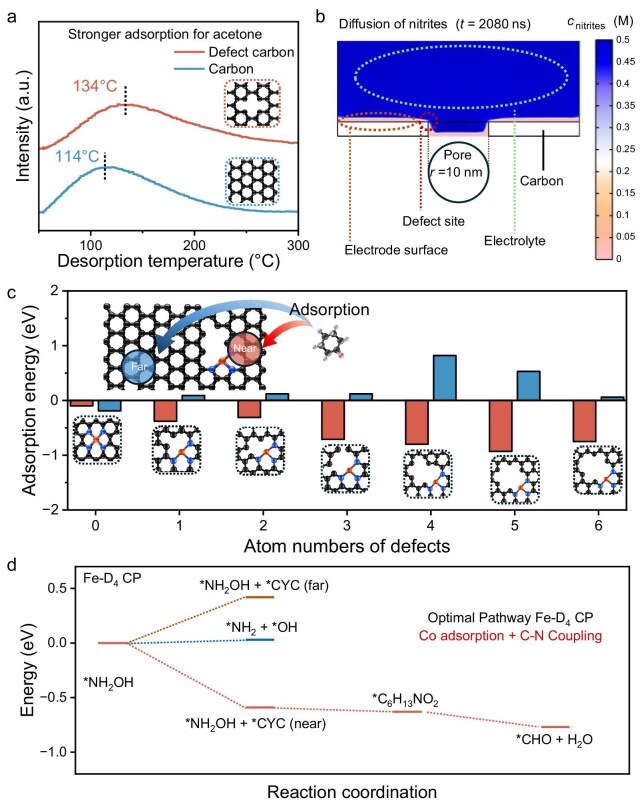
Theoretical calculations of Fe-defect catalytic pairs for near-stoichiometric conversion toward CHO. (a) Normalized acetone-TPD profiles of defect-poor carbon (blue line) and defect-rich carbon (red line). (b) Simulated surface NO_2_^−^ concentration near the defects (*r* = 10 nm) at a potential of −0.4 V. (c) Adsorption energy of CYC on the defect site (red column) and the site far away from the Fe site (blue column) for Fe-defect catalytic pairs with different defect sizes. (d) Free energy profiles of reaction pathways for the formed *NH_2_OH over Fe-D_4_ CP.

It is reported that carbon defects can facilitate the specific adsorption of carbonyl compounds; therefore we synthesized defect-rich carbon and defect-poor carbon (Figs S20 and S21). Acetone-TPD tests show that the acetone-desorption peak temperature on defect-rich carbon (134°C) is higher than that on defect-poor carbon (114°C), proving that carbon defects can promote the adsorption of carbonyl compounds (Fig. 4a) [38,39]. Besides, COMSOL simulations prove that the NO_2_^−^ concentration at defect sites is significantly higher than that at the electrode surface without defects under negative potential (Fig. 4b and Fig. S22). Hence, theoretically, the Fe-defect catalytic pair containing an isolated Fe site and accompanying defect has the potential for ensuring the co-adsorption of *NH_2_OH and *CYC during the electrocatalytic reductive coupling of NO_2_^−^ and CYC.

Based on the structure of Fe-defect catalytic pairs, we investigate the influence of defect sizes (ranging from one to six atoms) on CYC adsorption. DFT calculations show that, for a series of cases involving different defect sizes, the CYC adsorption on the Fe-defect catalytic pair gives priority over that on other sites (Fig. 4c, Fig. S23, and Table S6). Owing to the relatively stronger ability of *CYC adsorption, the Fe-defect catalytic pair, whose defect includes four atoms (defined as Fe-D_4_ CP), was chosen as the model structure for predicting the reaction pathway of electrocatalytic CHO synthesis based on Fe-D GSCs. The NO_2_RR pathway on the Fe-D_4_ CP follows Pathway 4→8 involving *NH_2_OH, which is similar to Fe SSCs (Fig. S24 and Table S7).

For the evolution of *NH_2_OH adsorbed on the Fe-D_4_ CP, the co-adsorption of CYC and NH_2_OH at the Fe-D_4_ CP has an absolute priority over the adsorption of CYC at other distant sites (Fig. 4d, Fig. S25, and Table S8). In addition, the free energy change of the subsequent C–N coupling is much lower than that of the *NH_2_OH reduction (Fig. 4d). Theoretically, at the Fe-defect catalytic pair, one NO_2_^−^ molecule and one CYC molecule can be converted to one CHO molecule. On the contrary, as to single Fe sites, *NH_2_OH may be excessively reduced to NH_3_, especially when *CYC is far away from *NH_2_OH. Hence, the Fe-D GSCs with few isolated Fe sites may be an ideal electrocatalyst for the near-stoichiometric conversion towards CHO. The difficulty in the synthesis of Fe-D GSCs is how to reduce the amounts of single Fe sites as far as possible.

### Synthesis and characterizations of Fe-D GSCs

**Figure 5.**
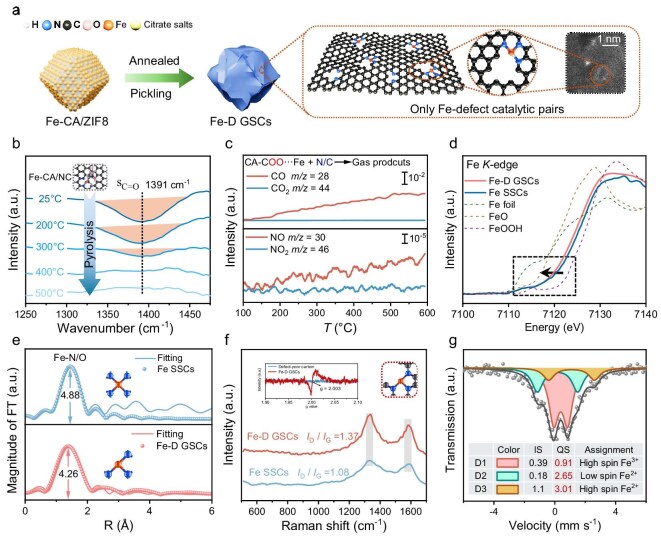
Synthesis and characterizations of Fe-D GSCs containing Fe-defect catalytic pairs. (a) Schematic diagram showing the synthesis strategy of Fe-D GSC (inset: the AC-STEM image of Fe-D GSC). (b) FT-IR spectra of Fe-CA/NC after hydrolysis at different temperatures. (c) MS response of CO (*m/z*: 28), CO_2_ (*m/z*: 44), NO (*m/z*: 30), and NO_2_ (*m/z*: 46) for the TGMS of Fe-CA/NC (argon atmosphere). (d) Normalized Fe K-edge XANES of Fe SSCs and Fe-D GSCs. (e) Fourier-transformed EXAFS in real-space (R-space) of Fe SSCs and Fe-D GSCs. (f) Raman spectra of the Fe SSCs and Fe-D GSCs (inset: EPR spectrum of Fe-D GSC). (g) Mössbauer spectra of Fe-D GSC at room temperature.

In order to reduce the amounts of single Fe sites, we synthesized Fe-D GSCs using ferrous-citrate complexes loaded on a zeolitic imidazolate framework-8 (ZIF-8) as precursors, and thereinto, accompanying-defects were *in-situ* constructed near isolated Fe sites to form the Fe-defect catalytic pair during pyrolysis (Fig. 5a and Figs S26–S28). Moreover, to acquire high-quality images for observing Fe-defect catalytic pairs, NH_3_-treated few-layer graphene (FG) was used as the carbon support [40]. Fe SSCs/FG and Fe-D GSCs/FG were synthesized using Fe phthalocyanine and ferrous-citrate complexes as precursors, respectively (Fig. S29). There are a few defects around single Fe sites for Fe SSCs, and more importantly, numerous single Fe sites are loaded on the non-defect layer of graphene (Fig. S30). Aberration corrected transmission electron microscopy (AC-STEM) images of Fe-D GSC show that there is always an accompanying defect close to each isolated Fe site (Fig. S31). Hence, the pyrolysis of ferrous-citrate complexes could ensure the formation of Fe-defect catalytic pairs in the Fe-D GSC.

*In-*/*ex-situ* spectra were further performed to investigate the evolution of ferrous-citrate complexes during the pyrolysis process. For the pyrolysis of ferrous-citrate complexes loaded on a carbon support (Fe-CA/NC), the FTIR signal of carboxylate ions decreased with increasing pyrolysis temperature (Fig. 5b and Fig. S32). Meanwhile, this pyrolysis process was accompanied by the formation of gaseous CO (*m/z* = 28) according to thermogravimetric mass spectrometry (TGMS) results (Fig. 5c). These results prove that oxygen atoms in the ferrous-citrate complexes can combine with carbon atoms to generate CO, which means that accompanying carbon defects are formed close to isolated Fe sites. Besides, TGMS also idenifies the formation of NO gas (*m/z* = 30) during the pyrolysis process, indicating that Fe-D GSCs possess a small amount of nitrogen defects (Fig. 5c).

Fe K-edge X-ray absorption near-edge structure (XANES) and extended X-ray absorption fine-structure (EXAFS) were performed to verify whether the nitrogen defects border on isolated Fe sites for Fe-D GSCs (Figs S33–S35). The absorption edge energy of Fe-D GSCs is slightly lower than that of Fe SSC, indicating that the Fe oxidation state in Fe-D GSCs is lower than that in Fe SSCs (Fig. 5d). This phenomenon might be caused by the accompanying defects in Fe-D GSCs. The least-squares EXAFS curve fitting results show that the coordination numbers for Fe-N paths in Fe-D GSCs (CN: 2.8) are nearly one coordination number less than that in Fe-SSCs (CN: 3.7), indicating that almost every isolated Fe site borders an accompanying N defect in Fe-D GSCs (Fig. 5e and Table S9).

Fe-D GSCs possess a higher *I*_D_/*I*_G_ value (1.37) than Fe-SSCs, proving that the graphitization degree of Fe-D GSCs is much lower than that (1.08) in Fe-SSCs (Fig. 5f). Electron paramagnetic resonance (EPR) spectra show that Fe-D GSCs are rich in carbon defects (g = ∼2.003) (Fig. 5f and Fig. S36). According to AC-STEM images of Fe-D GSCs, almost each Fe site borders on one nitrogen defect and several carbon defects (Fig. S31). According to the Mössbauer spectra, quadrupole splitting values of Fe species in Fe-D GSCs are much higher than those in traditional Fe SSCs, indicating that the electron density of Fe species in Fe-D GSCs exhibits a high asymmetry (Fig. S37) [41–45]. This specific characteristic of Fe-D GSCs should result from accompanying N/C defects in Fe-defect catalytic pairs. The highly asymmetry structure of Fe-defect catalytic pairs in Fe-D GSCs might facilitate reductive cross-coupling during electrocatalytic CHO synthesis.

### Near-stoichiometric conversion for electrocatalytic CHO synthesis

**Figure 6.**
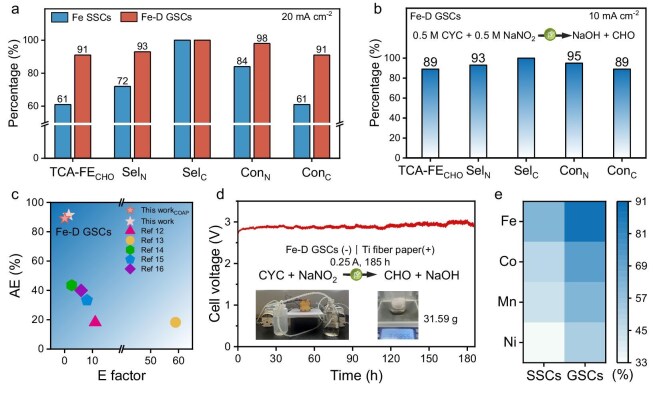
Near-stoichiometric conversion for electrochemical CHO synthesis over Fe-D GSCs. (a) TCA-FE_CHO_, Sel_N_, Sel_C_, Con_C_, and Con_N_ over Fe SSCs and Fe-D GSCs for electrochemical CHO synthesis from 0.5 M NaNO_2_ and 0.5 M CYC (electrolyte: 0.5 M Na_2_CO_3_) at theoretical coulomb amounts (1930 C). (b) TCA-FE_CHO_, Sel_N_, Sel_C_, Con_C_, and Con_N_ for the cathodic oxime-alkali process based on Fe-D GSCs (without the Na_2_CO_3_ electrolyte). (c) Comparison of E factors and atomic economy for electrochemical synthesis of CHO between this work and previous reports. (d) Stability tests of the Fe-D GSCs in the flow cell for 185 hours. (e) TCA-FE_CHO_ of different M SSCs and M-D GSCs for electrochemical CHO synthesis.

Interestingly, Fe-D GSCs show near-perfect performances for the electrocatalytic synthesis of CHO from CYC and NO_2_^−^ (stoichiometric feeding at the high concentration of 0.5 M), and both TCA-FE_CHO_ (91.3%) and selectivities (N_sel_: 93.2%; C_sel_: 100%) are almost up to the theoretical limit even at almost complete conversion (Fig. 6a and Figs S38–S40). The production rate of CHO in H-cells is 21.1 mg g_cat_^−1^ h^−1^ because the over-reduction of *NH_2_OH to NH_3_ will be more favorable in high current density. Furthermore, Fe-D GSCs could achieve the near-stoichiometric yields of CHO for 14 cycles, with the TCA-FE_CHO_ exceeding 88% for each cycle (Fig. S41). Fe-D GSCs can not only realize the near-stoichiometric conversion towards CHO, but also exhibit virtually unattenuated chemical stability (Fig. S42). Therefore, Fe-D GSCs satisfy the fundamental demands of industrialization for the electrocatalytic synthesis of CHO.

Under the inspiration of the mature chlor-alkali process, we developed an innovative cathodic oxime-alkali process (COAP) by using a mixed solution containing NaNO_2_ and CYC as cathodic electrolytes, in which high concentration NaNO_2_ and CYC can be electrocatalytically converted to high-purity products including sodium hydroxide (NaOH) and CHO (Fig. 6b and Fig. S43). The COAP system not only avoids the use of the extra electrolyte (i.e. Na_2_CO_3_), but also achieves the upgrading of Na^+^ anions to NaOH during electrocatalytic CHO synthesis. Due to the absence of the additional electrolyte (Na_2_CO_3_), the optimal current density of the COAP system is well below that of the system containing the Na_2_CO_3_ electrolyte (Fig. 6b). For the COAP system based on Fe-D GSCs, TCA-FE_CHO_, and yield_C/N_ can still reach 89% at 10 mA cm^−2^ so that this COAP system can achieve conversion from NaNO_2_ and CYC to NaOH and CHO at near-stoichiometric yields (Figs S43 and S44). The E factors (kg waste/kg product) and atom economy (AE, mol of product/sum of mol of starting materials) of this COAP system are extremely low compared with those systems reported in other studies (Fig. 6c and Table S10) [46].

Furthermore, the flow cell with a 25 cm^2^ electrode was used to increase the productivity of the cathodic oxime-alkali process. The cell voltage remained relatively stable for nearly 185 hours at a current of 0.25 A, and 31 g of CHO were synthesized at the isolated yield of 55.7% (Fig. 6d, Figs S45 and S46). CHO productivity is ∼1.5 mmol_CHO_ h^−1^, which has significant room for improvement [47–50]. The isolated yield is limited to the diffusion of organic molecules from the cathode to the anode. Besides, we developed a series of M-D SSCs via the accompanying defect strategy, and M-D GSCs achieve highly competitive performances and more excellent activities with respect to M SSCs for electrocatalytic CHO synthesis (Fig. 6e, Figs S47 and S48).

## CONCLUSION

This work reports a high atom-economy of 89% and a low E factor of 0.09 for the near-stoichiometric conversion to CHO from NO_2_^−^ and CYC at an initial concentration of 0.5 M on Fe-D GSCs. The proposed defect-accompanying strategy enables the precise synthesis of M-D GSCs via the pyrolysis of metal-citrate complexes loaded onto a carbon support. The liberation of gaseous carbon/nitrogen species and the strong coordination of citrates with metal ions ensures that almost every isolated metal site borders on accompanying defects to form M-D CP in M-D GSCs, thus eliminating single catalytic sites. Furthermore, an innovative cathodic oxime-alkali process is developed to avoid the waste of additional electrolytes via the direct conversion of CYC and NaNO_2_ into CHO and NaOH at near-stoichiometric yields. Subsequent research emphasis for the industrialization of electrocatalytic CHO synthesis is to solve the issue of diffusion of organic molecules through ion exchange membranes. More importantly, this work encourages researchers to develop other ERCR systems at near-stoichiometric yields via the design of metal-defect catalytic pairs in M-D GSCs.

## METHODS

Detailed methods can be found in the supplemental information.

## Supplementary Material

nwag206_Supplemental_File
