# Supplementary material for: Metal-defect pairs for near-stoichiometric electrocatalytic C–N coupling
Source: Natl Sci Rev. 2026 Apr 9;13(14):nwag206. doi: 10.1093/nsr/nwag206 (PMC13397535; doi:10.1093/nsr/nwag206)
Supplement: nwag206_Supplemental_File [file nwag206_supplemental_file.pdf]

Supplementary information

Metal-defect pairs for near-stoichiometric electrocatalytic C-N coupling

Yandong Wu[1,†], Wei Chen[1,†], Yuqin Zou[1,†], Jinbo Wang[1], Ruiqi Wang[1], Nan Hu[2,3], Qie Liu[1], Mengyi Qiu[1], Yimin Jiang[1], Mengwei Han[1], Ming Yang[1], Leitao Xu[1], Jiawang Chen[1], Chao Xie[1,4], Dongdong Wang[1], Yabin Xu[1], Yongmin He[1], Xuning Li[5], Liwu Fan[3], Xiang Gao[3] and Shuangyin Wang[1,*]

[1]State Key Laboratory of Chem/Bio-Sensing and Chemometrics, College of Chemistry and Chemical Engineering, Hunan University, Changsha 410082, China;
[2]Department of Mechanical and Aerospace Engineering, Princeton University, Princeton, New Jersey 08544, USA;
[3]State Key Laboratory of Clean Energy Utilization, Zhejiang University, Hangzhou 310027, China;
[4]College of Chemistry and Chemical Engineering, Institute of Interdisciplinary Studies, Hunan Normal University, Changsha 410081, China and [5]State Key Laboratory of Catalysis, Dalian Institute of Chemical Physics, Chinese Academy of Sciences, Dalian 116023, China
***Corresponding author**. E-mail: shuangyinwang@hnu.edu.cn
[†]Equally contributed to this work.

Experimental section

Materials

Sodium carbonate (Na_2CO_3), methanol (CH_3OH), N,N-dimethylformamide (DMF), Zinc nitrate hexahydrate ($Zn(NO_3)_2\cdot 6H_2O$), sulfuric acid (H_2SO_4, about 98%) are purchased from Sinopharm Chemical Reagent Co., Ltd. 2-methylimidazole, ferrous citrate, manganese citrate, cobalt citrate hydrate and nickel citrate are purchased from Macklin Co., Ltd. Fe nanoparticles are purchased from Xuzhou Jiechuang New Material Technology Co. These reagents are directly used without purification. Deionized water is used in all experiments.

Methods

Preparation of N-doped carbon materials

12 mmol $Zn(NO_3)_2\cdot 6H_2O$ was dissolved in 100 mL CH_3OH, and the mixture was added into the 60 mL CH_3OH solution with 48 mmol 2-methylimidazole. Afte aging for 24 hours, white precipitates (ZIF-8) are collected by extraction filtration and methanol washing. 1 g dried ZIF-8 powder was calcined at 950°C for 2 hours with a heating rate of 5°C/min to obtain N-doped carbon (NC).

Preparation of M Nps

6 mmol of metal nitrate hydrate (Co, Ni) were dissolved in the deionized water (30 mL). 50 mL 0.5 M Na_2CO_3 aqueous solution n was slowly added into the metal nitrate hydrate solution with vigorous stirring, obtaining the suspension. After stirring for 30 min, the suspension was poured into a Teflon-lined stainless-steel autoclave (100 ml). Then the Teflon-lined stainless steel autoclave was heated at 100 °C for 8 hours. After the Teflon-lined stainless steel autoclave cooled down naturally to room temperature, the obtained suspension was filtered and washed with deionized water for four times, then was transferred into the vacuum drying oven at 60°C for 12 hours. As-obtained powder was calcined at 600°C for 2 hours with a heating rate of 5°C/min under argon/hydrogen atmosphere to synthesize metal nanoparticles.

Preparation of Fe SSCs/FG

Firstly, multiple-layer graphene (FG) was calcined under NH_3 atmosphere for 2 hours at 400°C (a heating rate of 5°C min^{-1}). Then FG was spin coated with 50 µl 1000 ppb iron phthalocyanine solution. After calcining at 600°C for two hours, the Fe SSCs/FG were obtained.

Preparation of Fe-D GSCs/FG

Firstly, FG was calcined under NH_3 atmosphere for 2 hours at 400°C (a heating rate of 5°C min^{-1}). Then FG was spin coated with 50 µl 1000 ppb ferrous-citrate solution. After calcining at 600°C for two hours, the Fe-D GSCs/FG were obtained.

Preparation of M SSCs

1 g ZIF-8 powder was evenly dispersed in 50 mL ethanol via ultrasonic treatment for 60 min, and 10 mL *N*, *N*-dimethylformamide with 50 mg metal phthalocyanine (M-Pc; M = Fe, Co, Ni and Mn) was added into the mixture. After stirring vigorously for 24 hours, ZIF-8 powder adsorbing ferric citrate was collected by extraction filtration and ethanol washing. As-obtained powder was calcined at 950°C for 2 hours with a heating rate of 5°C/min. After naturally cooling down, as-obtained black powder was heated at 80°C for 24 hours in 80 mL 0.5 M H_2SO_4 to remove the inevitable metal nanoparticles. Finally, M SSCs were obtained after washing.

Preparation of M-D GSCs

1 g dried ZIF-8 powder was evenly dispersed in 50 mL ethanol via ultrasonic treatment for 30 min, and 1 mL 50 mg/mL metal salt solution (ferrous citrate, manganese citrate, cobalt citrate hydrate and nickel citrate. After stirring vigorously for 24 hours, ZIF-8 powder adsorbing metal salts (AMS/ZIF-8) was collected by extraction filtration and ethanol washing. 1 g dried AMS/ZIF-8 was calcined at 950°C for 2 hours with a heating rate of 5°C/min. After naturally cooling down, as-obtained black powder was heated at 80°C for 24 hours in 80 mL 0.5 M H_2SO_4 to remove the inevitable metal nanoparticles. Finally, the M-D GSCs were obtained after washing.

Characterizations

Morphologies and crystalline structures of prepared catalysts were identified by scanning electron microscope (SEM, Hitachi S-4800, Hitachi Corporation, Japan) and spherical aberration corrected transmission electron microscope (STEM, Themis Z-3.2, Thermo scientific, Netherlands). Catalyst surface species was characterized by X-ray photoelectron spectroscopy (XPS, Axis Supra, Kratos Company, England). The Fe K-edge X-ray absorption spectroscopy was recorded using Taiwan Photon Source (TPS) Quick-scanning X-ray absorption spectroscopy beamline 44A1 at National Synchrotron Radiation Research Center (NSRRC; Hsinchu). Transmission mode using an ion chamber detector was carried out in the XAS measurement. The XAS spectra were calibrated using pure cobalt metal foil. All XAS spectra were measured at the oscillating frequency of 1 Hz for 2 min. We measured the spectra 240 times and then took the average of the XAS spectra to increase the S/N ration. All XAS spectra were aligned, merged, deglitched, and normalized using the Athena module (version number: 0.9.26) implemented in the IFEFFIT software packages. The confocal Raman microscope (Alpha300R, WETEC, Germany) equipped with a 50x objective and a 600 grooves/mm grating, was used to measure Raman spectra. It employed a 633 nm excitation laser with a power of 110 mW. The carbon defect was identified by EPR (JES-FA 200, JEOL Ltd., Tokyo, Japan). The room temperature Fe Mössbauer spectra and operando Fe Mössbauer measurements were carried out with a proportional counter and a Topologic 500A spectrometer with ^{57}Co (Rh) as a γ-ray radioactive source.

Electrocatalytic measurement

5 mg catalysts were evenly dispersed in 950 μl isopropanol via ultrasonic treatment for 60 min, and 50 μl Nafion (5%) was added into the suspension. Then, 200 μl catalyst ink was dropped on 1 cm × 1 cm treated carbon paper (calcined at 400°C for 16 hours with a heating rate of 5°C/min), and dried naturally to obtain the working electrode with the catalyst loading of 1 mg cm^{-2}. Without special instructions, linear sweep voltammetry and electrolysis tests used 1 cm × 1 cm treated carbon paper as the working electrode.

Linear sweep voltammetry measurement was performed in a three-electrode system (CHI630E, CH Instruments) with a scanning rate of 5 mV/s. Carbon rod and mercuric oxide electrode (Hg/HgO) were used as the counter electrode and reference electrode. Without special instructions, the electrolyte was 0.5 M Na_2CO_3 solution. The RHE calibration was measured using platinum gauze electrode as work electrode in the hydrogen-saturated electrolyte solution. All potential measured was calibrated to the reversible hydrogen electrode using the following equation: $E_{RHE} = E_{test} + 0.098\ V + 0.059\times pH$. The electrolysis experiments in this work all used the constant

current model for further flow continuous production.

Membrane electrode assembly fabrication and flow cell test

The anion-exchange membrane (Grade RT membrane) was purchased at Suzhou Sinero technology co., LTD. The membrane was immersed in 1 M KOH for at least 24 h before using. The cathode electrode is fabricated as follows. 70 mg electrocatalysts were dispersed in isopropanol solution by sonication for 1 h, then add 140 mg 5% Nafion solution as a binder. The catalyst was sprayed onto the hydrophilic carbon paper (6 cm × 6 cm) by ultrasonic spraying. The loading amount of Fe-D GSCs on the carbon paper was controlled to be about 2.0 mg cm^{-2}. The anode electrode was titanium fiber paper. The reaction area in the flow cell is 5 cm × 5 cm. The flow rate is 6 mL min^{-1} both at the anode side and cathode side. Reasonably, due to the high concentration of CYC, the diffusion of CHO and CYC still exist during the long-time electrolysis, leading to an obvious decrease in the isolated yield. Therefore, 1 L 0.55 M $NaNO_2$ with 0.5 M CYC aqueous solution is used for short-time electrolysis.

Product Analysis

The concentration of organic products (cyclohexanone, cyclohexanone oxime and cyclohexanol) are detected via gas chromatography (GC) with external method. GC samples were prepared as follows: (1) 10 ml electrolyte after electrolysis was extracted with 10 mL ethyl acetate for five times; (2) Before GC analysis, the ethyl acetate solution must be filtered.

The concentration of nitrites ($NaNO_2$) and ammonia (NH_3) are identified with UV-visible spectroscopy. The quantification of NO_2^- concentration is obtained by N-(1-naphthyl)-ethylenediamine dihydrochloride spectrophotometric method: (1) 5 mL treated electrolyte (100 ul electrolyte was added into 19.9 mL deionized water) and 0.1 mL color reagent (the mixture of 4.0 g p-aminobenzene sulfonamide, 0.2 g N-(1-Naphthyl) ethylenediamine dihydrochloride, 50 mL ultrapure water and 10 mL 85wt% phosphoric acid) were added into a 5 mL glassy bottle; (2) The solution was mixed uniformly and the absorption intensity at a wavelength of 538 nm was recorded after reacting in a dark environment for 20 min.

The quantification of NH_3 concentration is conducted by the indophenols blue method : (1) 2 mL treated electrolyte (100 μL electrolyte was diluted with 19.9 mL deionized water), 2 mL A solution, 1 mL B solution and 0.2 mL C solution were added into a 5 mL glassy bottle (A: 1 M NaOH solution containing 5wt% salicylic acid and 5wt% sodium citrate; B: 0.05 M NaClO solution; C: 1wt% sodium nitroferricyanide solution); (2) The solution was mixed uniformly and the absorption intensity at a wavelength of 662 nm was recorded after reacting in a dark environment for 120 min.

The C/N conversion rate, C/N selectivity, C/N yield and faradaic efficiency for the direct electrosynthesis of oxime are as follows:

C conversion rate = n (formed CHO) / n (initial cyclohexanone)

N conversion rate = n (formed CHO) + n (formed NH_3) / n (initial NO_2^-)

C selectivity = n (formed CHO) / (n (formed CHO)+n (formed cyclohexanol))

N selectivity = n (formed CHO) / (formed CHO)+n (formed NH_3))

C yield = C selectivity×C conversion rate

N yield = N selectivity×N conversion rate

$TCA\text{-}FE_{CHO} = e \times n\ (\text{formed CHO}) \times F / (I \times t)$

Faradaic efficiency (NH_3) = $e \times n$ (formed NH_3)$\times F / (I \times t)$

where e is the number of electrons transferred (e = 4 for the electrosynthesis of CYO; e = 6 for the electrosynthesis of NH_3), F is the Faraday constant (96500 C mol^{-1}), n is the amount of product (in moles).

It should be noted that the high concentration of CYC in this work must result from the diffusion of CYC and CHO to the anode cell. In the anode side, CHO tends to be oxidized to nitric oxide (NO) and CYC, leading to difficulties in the measurement of the concentration of CHO in the anode. Meanwhile, we tested the total concentration at the anode and cathode cells, and the total concentration of CHO and CYC at the anode are all below 50 mM. Therefore, we used the ratio of CHO at the cathode side to estimate that at the anode side. Indicators in this work were all calculated as above methods.

Theoretical calculation

Metal single site and metal-defect catalytic pair are placed in a graphene layer at a vertical distance of 20 Å. The vacuum layer between periodically repeated slabs was set as 15 Å to avoid interactions among slabs. All the DFT calculations were performed via using Vienna Ab-initio Simulation Package (VASP) with the projector augmented-wave approach for the interaction between the ionic core and valence electrons[1-3]. The Perdew-Burke-Ernzerhof (PBE) functional was used for correlation energy density function and core-valence interactions, respectively[4]. The cutoff energy of 450 eV was applied and the DFT-D3 method was used to calculate van der Waals interactions[5,6]. The convergence criteria for energy tolerance and maximum force tolerance were set to 0.03 eV $Å^{-1}$ and 1×10^{-5} eV, respectively. The Brillouin zone was sampled at Gamma point for geometry optimization. DFT + U method was used for series of metal single site and metal-defect catalytic pair. The calculation is performed at the potential of 0 V (versus RHE). The free energy change was acquired by:

$$\Delta G = \Delta E + \Delta E_{ZPE} - T\Delta S$$

where ΔE is the difference of electronic energy. ΔS is the entropy (S) variation, ΔE_{ZPE} is the change of zero-point energy (ZPE), T is the temperature (T = 298.15 K). The transition state was located using the climbing image nudged elastic band (CI-NEB) and Dimer method. A stretching frequency analysis was also performed to verify whether a transition state is associated with a single imaginary frequency. Adsorption energy (E_{ads}) can be obtained according to the following equation:

$$E_{ads} = E_{AB} - E_A - E_B$$

where E_{AB} is the energy of system AB after substance A is adsorbed on substrate B. E_A is the energy of substance A and E_B is the energy of substrate B. E_{ads} represents the total energy of the system after the adsorption of intermediate molecules on the surface after molecular dynamics calculations and structural relaxation under this model, which can be used to evaluate the strength

of adsorption on the surface.

DEMS measurement

Online DEMS (QAS100, Linglu Instruments Co., Lt, China) was carried out in the DEMS system. The working electrode substrate is a 50 nm gold-sputtered PTFE membrane. The ink of electrocatalysts consists of 5 mg electrocatalysts, 600 μl isopropanol, 300 μl ultrapure water and 100 μl Nafion solution. Then, the catalysts were dropped on the Au film with a mass loading of 0.75 mg cm^{-2} and dried naturally. The gaseous products and electrode-adsorbed intermediates during eNO_2RR were pumped into a mass spectrometer detector. The MS signals were collected during the linear sweep voltammetry processes at a scan rate of 5 mV s-1. 0.5 M Na_2CO_3 with 0.5 M $NaNO_2$ was chosen as the electrolytes. The saturated Ag/AgCl electrode and a Pt wire were used as the reference electrode and counter electrode, respectively.

Thermogravimetry-mass spectrometry measurement

A simultaneous thermal analyzer (Mettler Toledo, model TGA/SDTA 851e) coupled online with a quadrupole mass spectrometry (Pfeiffer Vacuum, model Thermostar GSD301T3) was applied for the TG–MS analysis on the thermal decomposition process. The gases generated in the simultaneous thermal analyzer were blow into the MS detector to identify the reaction during the pyrolysis. The catalysts sample in this experiment were 10 mg N-C adsorbing ferric citrate powder. The pyrolysis experiment was performed from 30°C from 600°C under argon flow (50 mL min^{-1}) with a heating frate of 10°C min^{-1}.

COMSOL simulation

Supplementary Fig. 22a show the schematic diagram of the simulation region in cylindrical coordinates, where Ω represents the bulk phase, S represents the surface. The subscripts l and s denote liquid and solid, respectively, while L, T, R, and B represent the left, top, right, and bottom boundaries, respectively, with i indicating the interface.

In the liquid phase, we consider the governing equation as the Nernst-Planck-Poisson equation:

$$\frac{\partial C_i}{\partial t} + \nabla(-D_i\nabla C_i - z_i\mu_i F C_i \nabla\phi + uC_i) = 0$$

$$\frac{\partial \phi_l}{\partial t} + \nabla^2\phi_l = -\frac{\sum_i z_i e_i}{\epsilon}$$

where C is the concentration, t is time, D is the diffusion coefficient, z is the charge number, F is the Faraday constant, μ is the ionic mobility, ϕ is the electric potential, u is the velocity, e is the elementary charge, and ϵ is the dielectric constant. The subscripts i, l, and s represent different chemical species, the liquid phase, and the solid phase, respectively.

In the solid phase region, we need to calculate the transient potential distribution under a fixed potential, with the governing equation

$$\frac{\partial \phi_s}{\partial t} + \nabla^2\phi_s = 0$$

This simulation region involves five boundary conditions:

$$\frac{\partial \phi}{\partial n} = 0 \;\&\; \frac{\partial C_i}{\partial n} = 0 \; at \; S_L \;\&\; S_R$$

$$\frac{\partial \phi}{\partial n} = 0 \ \& \ C_i = C_{i,t} \ at \ S_T$$

$$\phi_l = \phi_s \ \& \ \frac{\partial C_i}{\partial n} = R_i \ at \ S_i$$

$$\phi_s = \phi_{s,b} \ at \ S_B$$

where n represents the unit normal vector perpendicular to the surface and R_i denotes the source term for surface adsorption reactions. Assuming an isothermal adsorption model, this is written as:

$$R_i = k_{i,d} C_{i,sat} \theta - k_{i,a} C_{i,A} C_{i,sat} (1 - \theta)$$

where k is the desorption/adsorption rate, θ is the surface coverage ratio, and subscripts d, a, and sat represent desorption, adsorption, and saturation, respectively.

Geometry: The pore radius R is considered to be 5, 10, 20, and 40 nm. The liquid phase region has a height of 50 nm with $\epsilon_r = 1$, the solid phase region has a thickness of 20 nm with $\epsilon_r = 3$, and the horizontal width is 4R.

Diffusion coefficients are

$$D_{CYC} = 1.3 \times 10^{-9} \text{ m}^2\text{s}^{-1}$$

$$D_{Na^+} = 1.33 \times 10^{-9} \text{ m}^2\text{s}^{-1}$$

$$D_{NO_2^-} = 1.9 \times 10^{-9} \text{ m}^2\text{s}^{-1}$$

$$D_{CO_3^{2-}} = 0.923 \times 10^{-9} \text{ m}^2\text{s}^{-1}$$

Which enables to give the Ionic mobility according to the Einstein equation as

$$\mu_i = \frac{D_i z_i e}{k_B T}$$

Initial conditions:

$$C_{CYC} = C_{Na^+} = C_{NO_2^-} = C_{CO_3^{2-}} = 0M, \phi = 0V$$

Boundary conditions:

$$C_{CYC} = 0.5 \ M, C_{Na^+} = 1.5 \ M, C_{NO_2^-} = 0.5 \ M, C_{CO_3^{2-}} = 0.5 \ M \ at \ S_T$$

$$\phi_{s,b} = -0.4 \ V \ at \ S_B$$

The numerical solution to this equation is achieved using the COMSOL Multiphysics software. Both the grid and time dependence were verified using an extremely fine grid and a time step of 0.1 ns.

SUPPLEMENTARY FIGURES

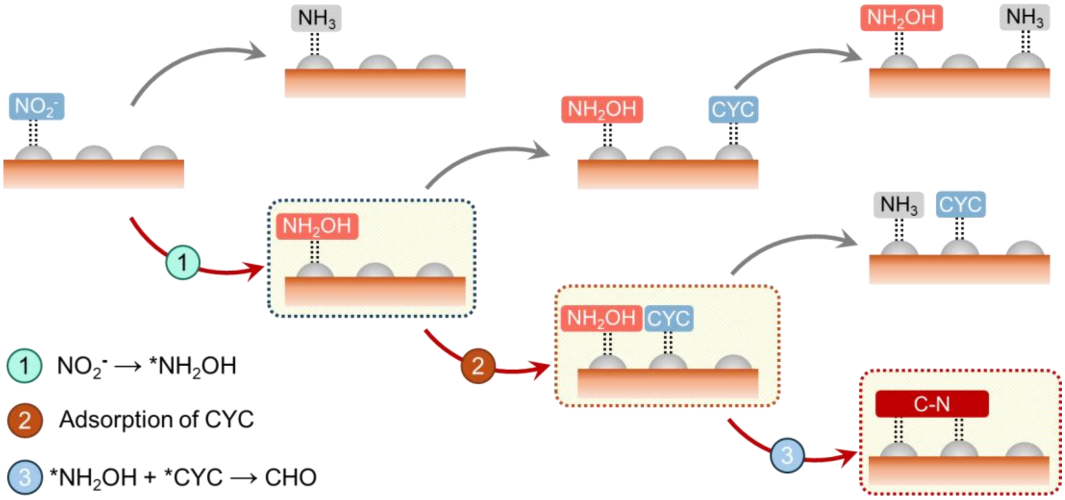

Figure S1. Schematic diagrams for the reductive C-N coupling of nitrites and CYC towards CHO.

Electrocatalytic synthesis of CHO from NO_2^- and CYC consists of the following three processes: (1) Selective electrocatalytic reduction of NO_2^- to $*NH_2OH$; (2) Adsorption of CYC adjacent to existing $*NH_2OH$; (3) C-N coupling of $*NH_2OH$ and *CYC towards CHO (Figure S1). For Process 1, electrocatalysts should facilitate the selective electrocatalytic reduction of NO_2^- to $*NH_2OH$ instead of other NO_2RR pathways, which can lead to the formation of by products, i.e., NH_3. In Process 2, $*NH_2OH$ tends to be over-reduced to NH_3 when *CYC is far away from $*NH_2OH$. The C-N coupling towards CHO only occurs at the co-adsorption of NH_2OH and CYC. Finally, the C-N coupling must give priority over the over-reduction of $*NH_2OH$.

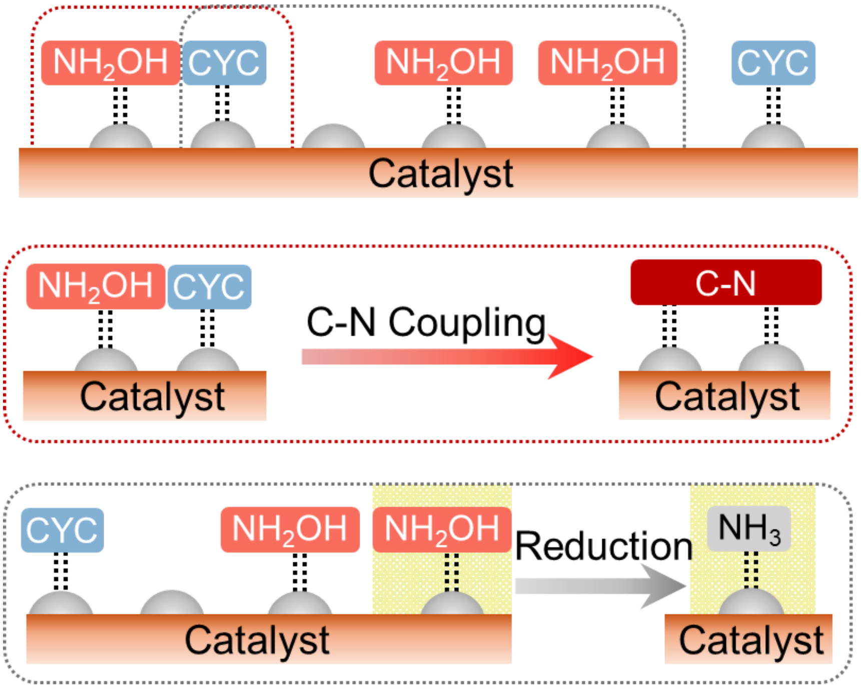

Figure S2. Schematic diagrams of how the uncertainty of CYC adsorption affects the selectivity of CHO.

The uncertainty of CYC adsorption directly affects the distribution of *CYC on the catalyst surface. When *CYC is close to the formed *NH_2OH, *CYC can capture *NH_2OH for the subsequent C-N coupling towards CHO. On the contrary, *CYC is far away from the formed *NH_2OH, the space distance prevents the C-N coupling of *CYC and *NH_2OH, thus leading to the over-reduction to NH_3.

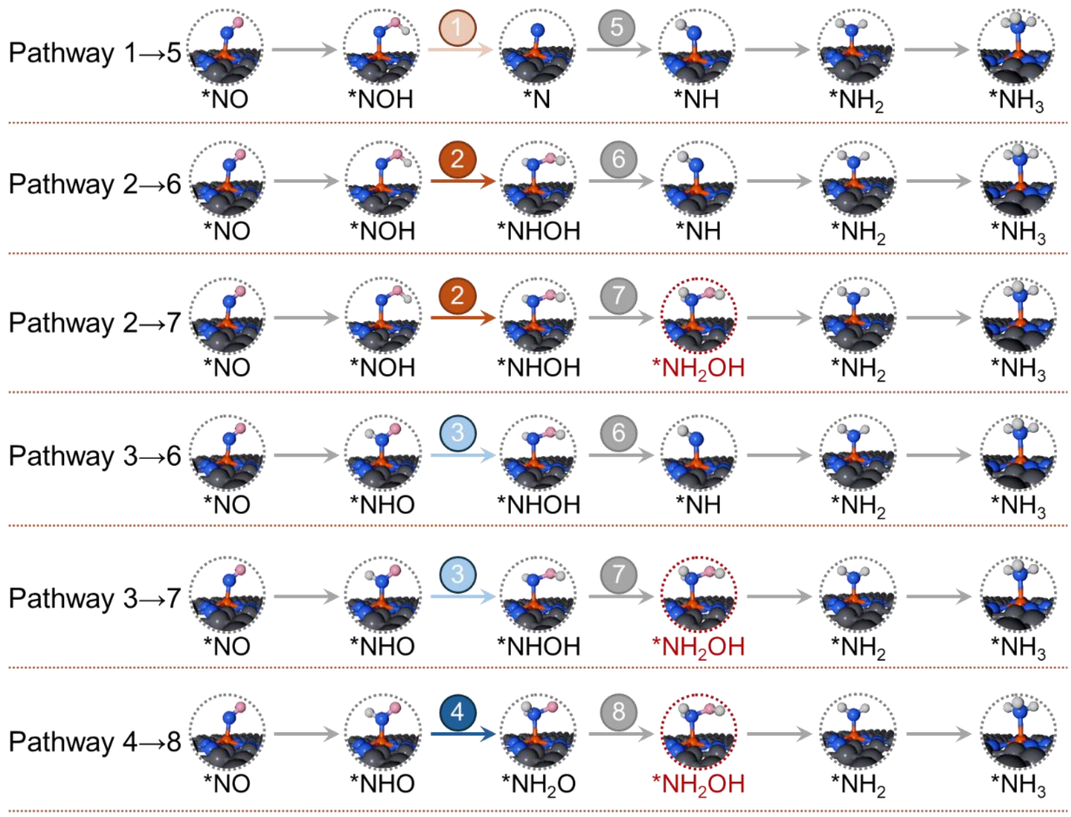

Figure S3. Schematic diagrams of reaction pathways for NO_2RR.

Reaction pathways for NO_2RR are divided into six types according to the reaction Step 1-8 (Step 1 *NOH→*N (light brown), Step 2 *NOH→*NHOH (brown), Step 3 *NHO→*NHOH (light blue), Step 4 *NHO→*NH_2O (blue), Step 5 *N→*NH, Step 6 *NHOH→*NH, Step 7 *NHOH→*NH_2OH, Step 8 *NH_2O→*NH_2OH). The reaction pathways are named according to the different steps in Supplementary Fig. 3.

Pathway 1→5: NO_2^-→*NO→*NOH→*N→*NH→*NH_2→*NH_3

Pathway 2→6: NO_2^-→*NO→*NOH→*NHOH→*NH→*NH_2→*NH_3

Pathway 2→7: NO_2^-→*NO→*NOH→*NHOH→*NH_2OH→*NH_2→*NH_3

Pathway 3→6: NO_2^-→*NO→*NHO→*NHOH→*NH→*NH_2→*NH_3

Pathway 3→7: NO_2^-→*NO→*NHO→*NHOH→*NH_2OH→*NH_2→*NH_3

Pathway 4→8: NO_2^-→*NO→*NHO→*NH_2O→*NH_2OH→*NH_2→*NH_3

Pathways 2→7, 3→7, 4→8 involves the formation of *NH_2OH, while Pathways 1→5, 2→6, 3→6 are not favorable for the enrichment of *NH_2OH. Therefore, only the electrocatalysts following Pathways 2→7, 3→7, 4→8 can realize the near-stoichiometric conversion towards CYO. Considering that the key intermediates *NO is an inevitable intermediate during NO_2RR, it was chosen as the start point for the DFT calculations in the manuscript.

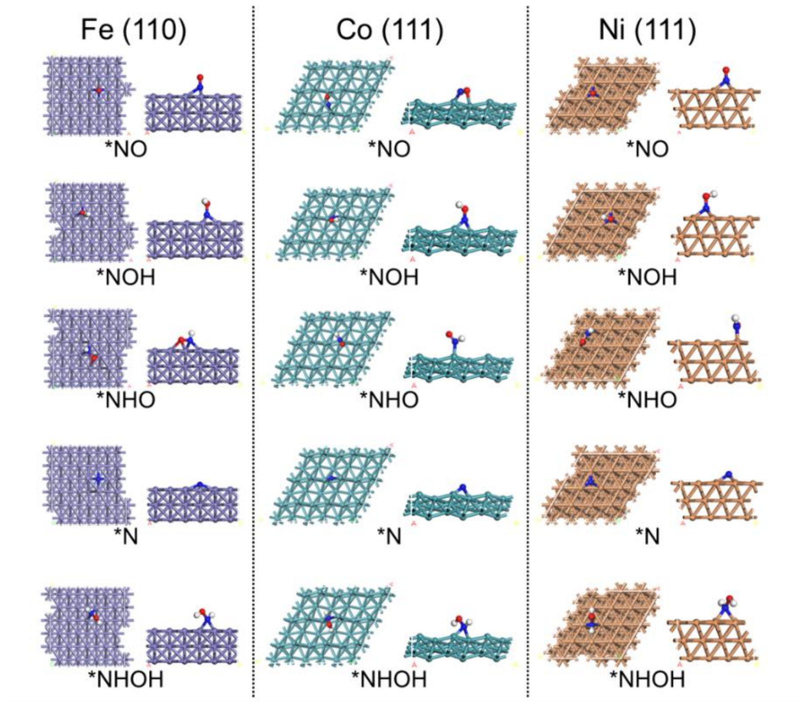

Figure S4. DFT structural models for NO_2RR on Fe (110), Co (111) and Ni (111).

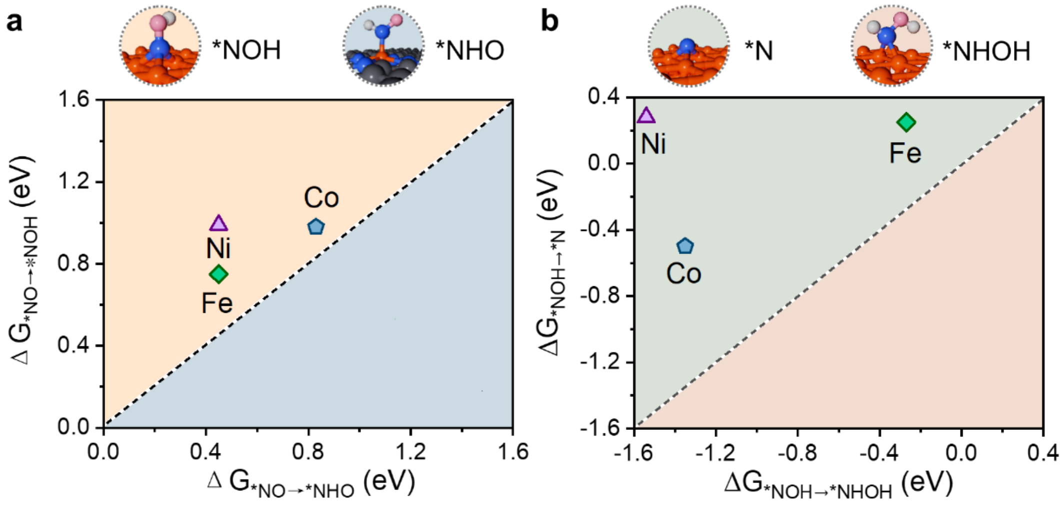

Figure S5. a, Comparison of the bifurcation of *NO→*NHO and *NO→*NOH reaction pathways over Fe Nps, Ni Nps and Co Nps. **b,** Comparison of the bifurcation of *NOH→*NHOH and *NOH→*N reaction pathways over Fe Nps, Ni Nps and Co Nps.

*NO is the inevitable intermediate for NO_2RR on almost all electrocatalysts so that *NO is chosen as the starting point in this work. In Supplementary Fig. 5, as to metal Nps, the hydrogenation from *NO to *NOH is more favorable than that to *NHO. Then the deoxidation to *N takes precedence over the hydrogenation to *NHOH over Fe (110), Ni (111) and Co (111).

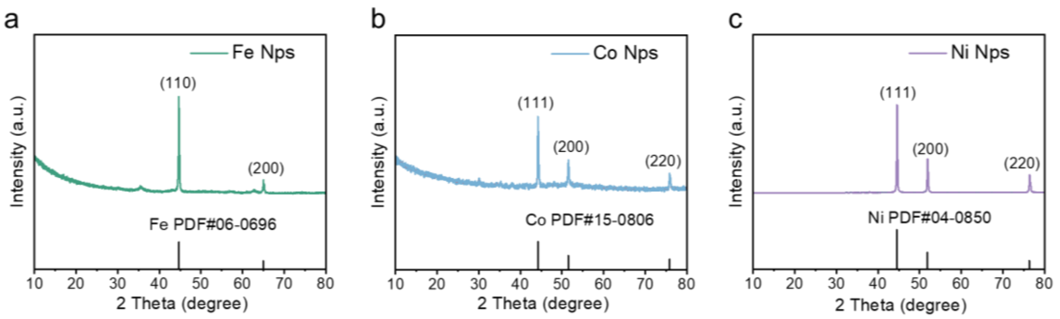

Figure S6. XRD patterns for Fe Nps **(a)**, Co Nps **(b)**, and Ni Nps **(c)**.

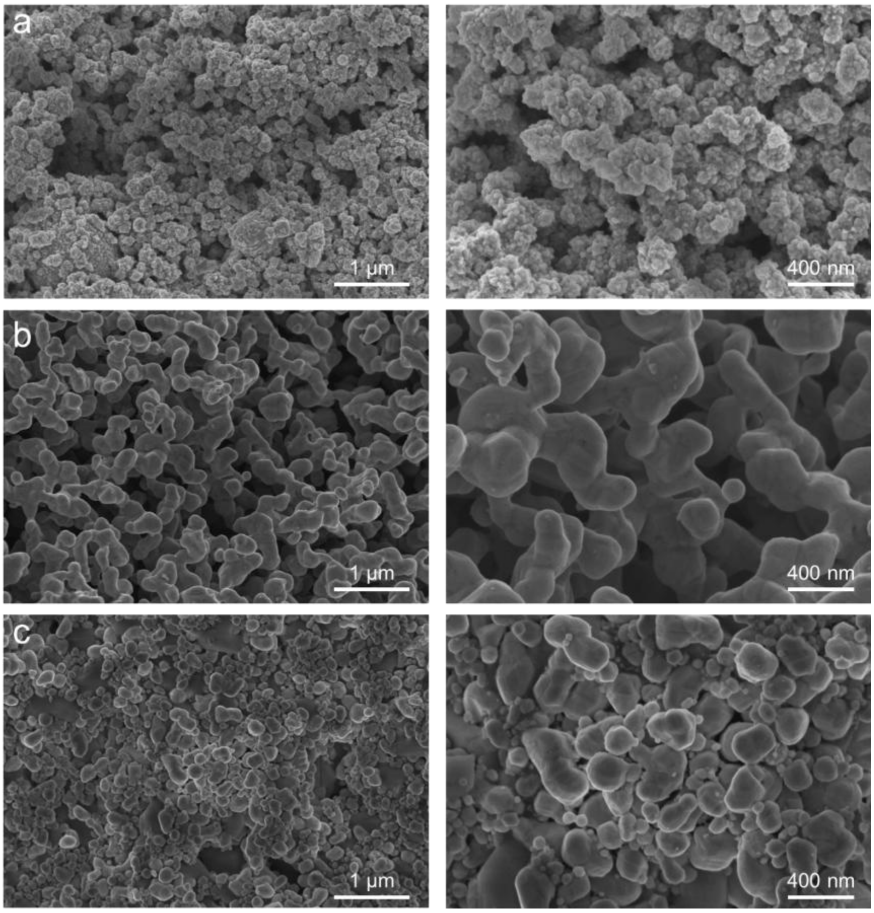

Figure S7. SEM images for Fe Nps **(a)**, Co Nps **(b)** and Ni Nps **(c)**.

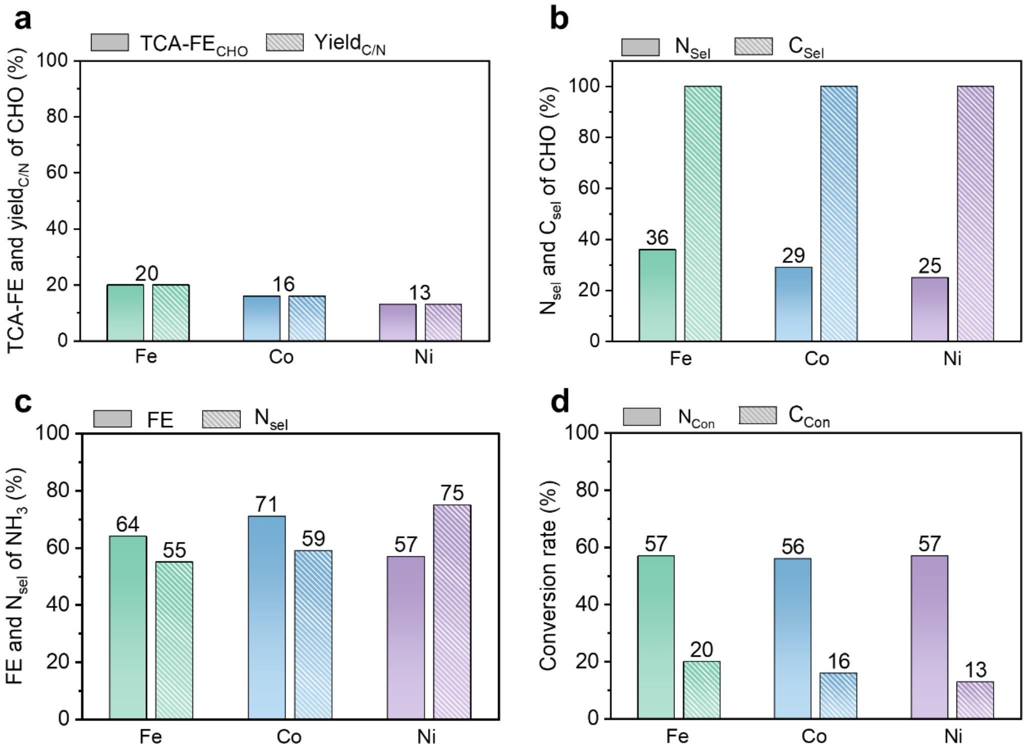

Figure S8. TCA-FE$_{CHO}$ & yield$_{C/N}$ of CHO **(a)**, N selectivity & C selectivity **(b)**, Faradaic efficiency & N selectivity of NH_3 **(c)** and N conversion rate & C conversion rate **(d)** of electrocatalytic synthesis of CHO over metal Nps electrocatalysts (Fe, Co, Ni). The constant electrolysis was all performed in 10 mL 0.5 M Na_2CO_3 with 0.5 M $NaNO_2$ and CYC at 20 mA cm^{-2} for 1930 C.

Although Fe electrocatalysts show the highest N selectivity of CHO (36%) and lowest N selectivity of NH_3 (55%), the TCA-FE$_{CHO}$ & yield$_{C/N}$ of CHO are extremely low due to the side reaction for NH_3 production. Therefore, metal Nps are not suitable sites for the near-stoichiometric conversion towards CHO.

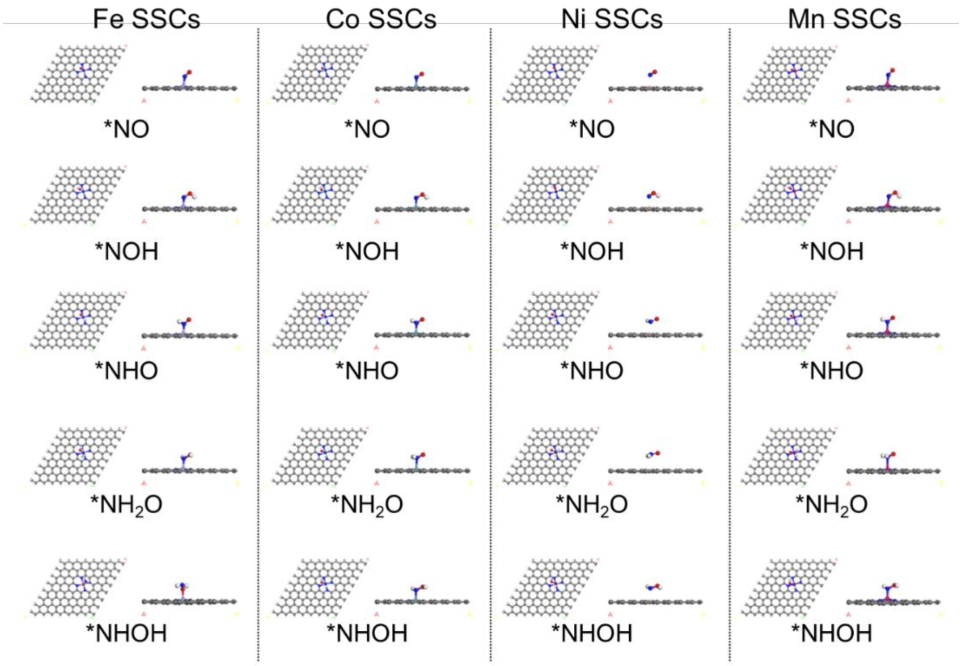

Figure S9. DFT structural models for NO_2RR on Fe (110), Co (111) and Ni (111).

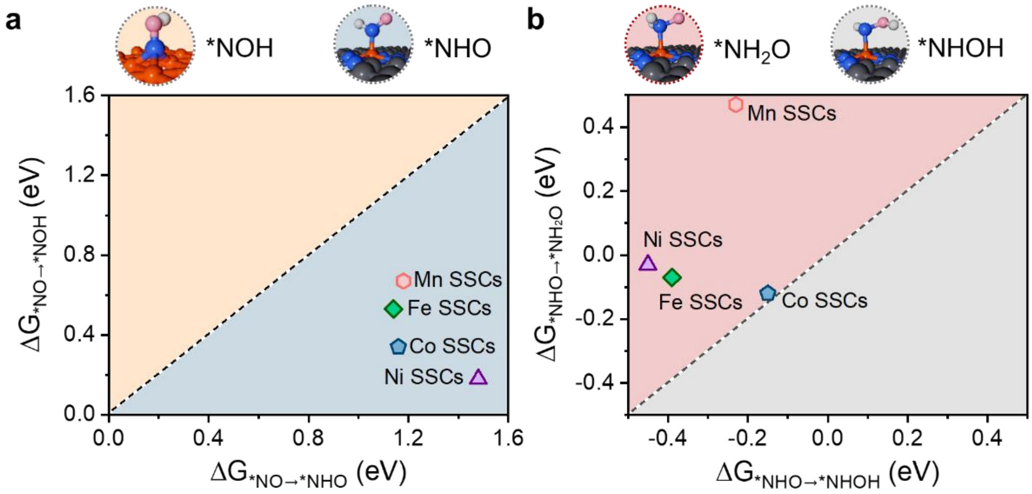

Figure S10. a, Comparison of the bifurcation of *NO→ *NHO and *NO → *NOH reaction pathways over Fe SSCs, Co SSCs, Ni SSCs and Mn SSCs. **b,** Comparison of the bifurcation of *NOH→ *NHOH and *NOH → *NH_2O reaction pathways over Fe SSCs, Co SSCs, Ni SSCs and Mn SSCs.

Series of M SSCs (M=Mn, Ni, Co, Fe) were all synthesized via an impregnation-adsorption method, and metal phthalocyanines (MPS) were used as the metal-precursors to maintain the M-N_4 structure in our DFT models. In this way, zeolitic imidazolate framework-8 (ZIF-8) was used as support to adsorb more MPS to form more active sites. The hydrogenation from *NO to *NHO is more favorable than that to *NOH over series of M SSCs (M=Mn, Ni, Co, Fe). Then the hydrogenation to *NH_2O takes precedence over the hydrogenation to NHOH, thus efficiently promoting the formation of *NH_2OH.

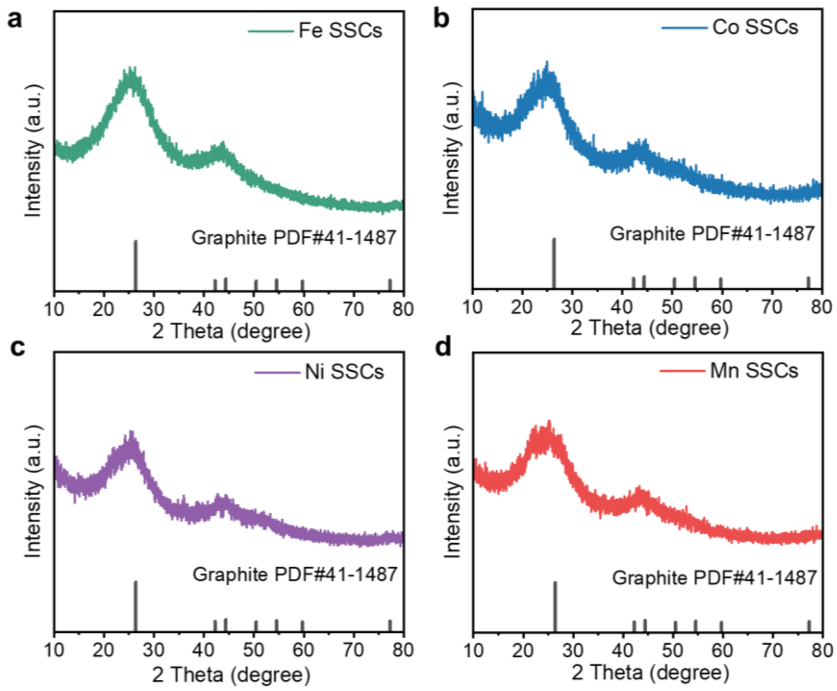

Figure S11. XRD patterns for Fe SSCs **(a)**, Co SSCs **(b)**, Ni SSCs **(c)** and Mn SSCs **(d)**.

There are no obvious signals of metal nanoparticles or metal oxide nanoparticles on series of M SSCs.

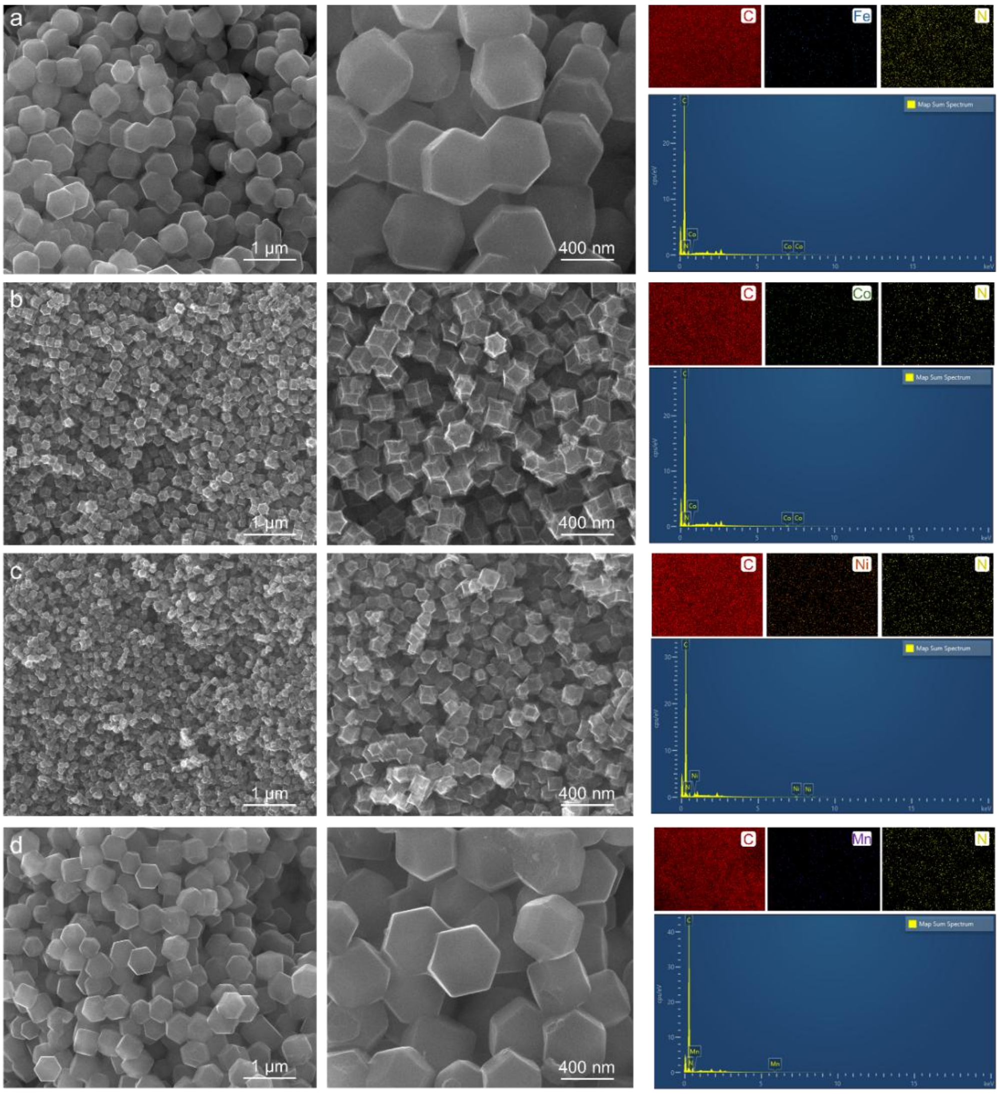

Figure S12. SEM images for Fe SSCs **(a)**, Co SSCs **(b)**, Ni SSCs **(c)** and Mn SSCs **(d)**.

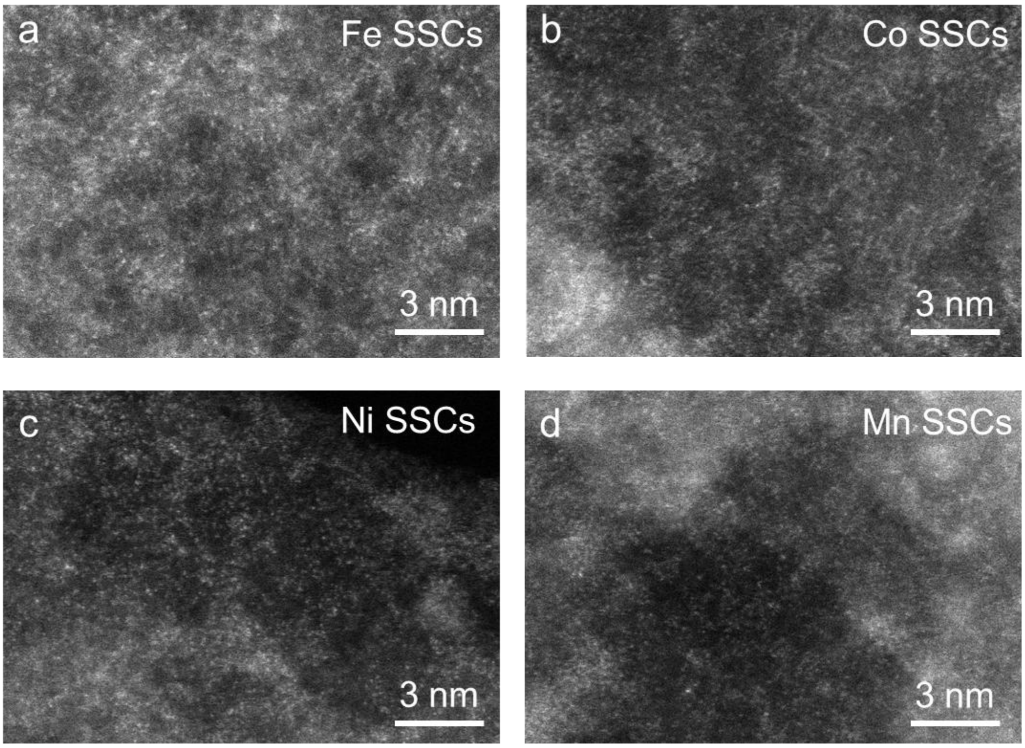

Figure S13. AC-STEM images for Fe SSCs **(a)**, Co SSCs **(b)**, Ni SSCs **(c)** and Mn SSCs **(d)**. These images prove that there are no obvious metal clusters or nanoparticles.

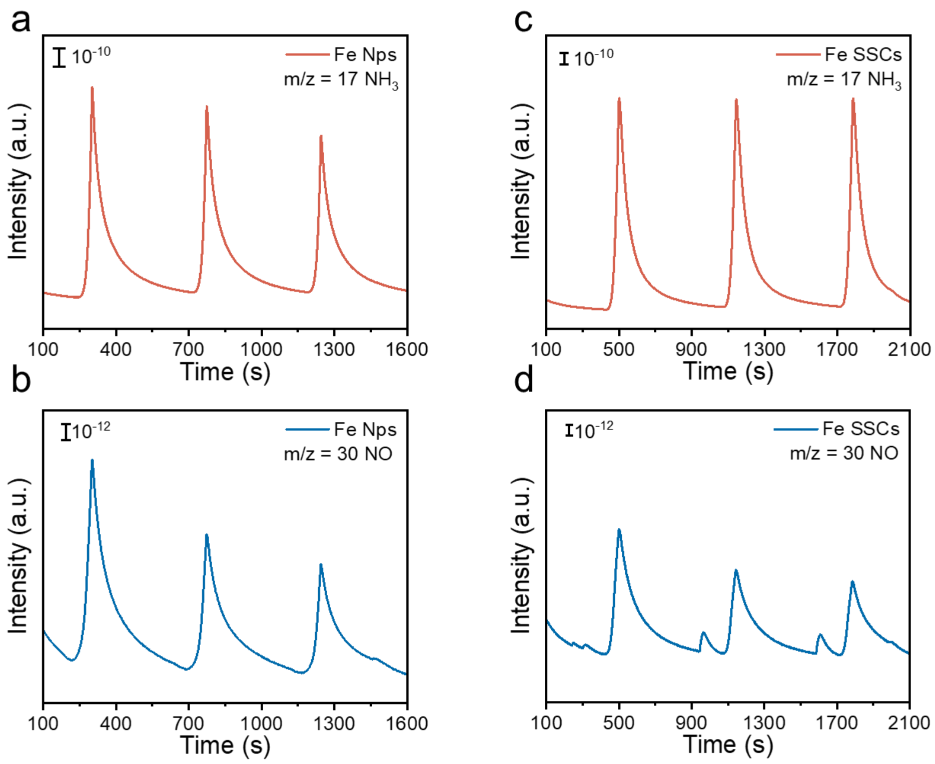

Figure S14. Real-time profiles corresponding to the generation of NH_3 (m/z = 17) and NO (m/z = 30) during the NO_2RR over Fe Nps and Fe SSCs electrocatalyst in differential electrocatalytic mass spectrometry (DEMS).

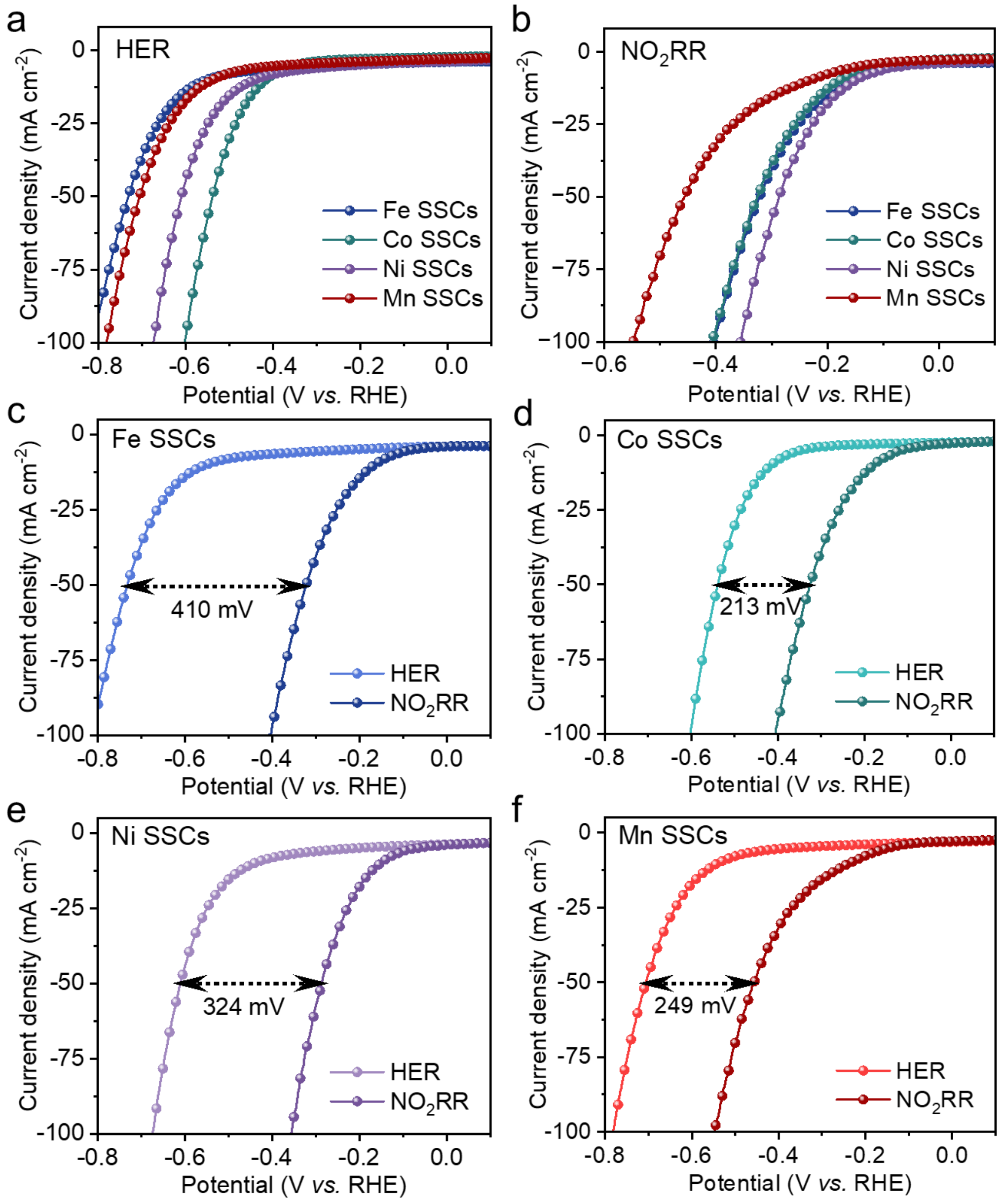

Figure S15. Linear sweep voltammetry (LSV) curves of HER **(a)** and NO_2RR **(b)** over M SSCs electrocatalysts (M= Fe, Co, Ni, Mn) at a scan rate of 5 mV/s. LSV curves of HER and NO_2RR over Fe SSCs **(c)**, Co SSCs **(d)**, Ni SSCs **(e)** and Mn SSCs **(f)**.

To evaluate the differences of reaction potentials between NO_2RR and HER, we choose the differences of reaction potentials at 50 mA cm^{-2} to screen the suitable isolated metal site. The differences of reaction potentials between NO_2RR and HER on Fe SSCs, Co SSCs, Ni SSCs and Mn SSCs are 410 mV, 213 mV, 324 mV and 249 mV. Therefore, Fe-SSCs have the biggest difference (410 mV) of reaction potentials between NO_2RR and HER.

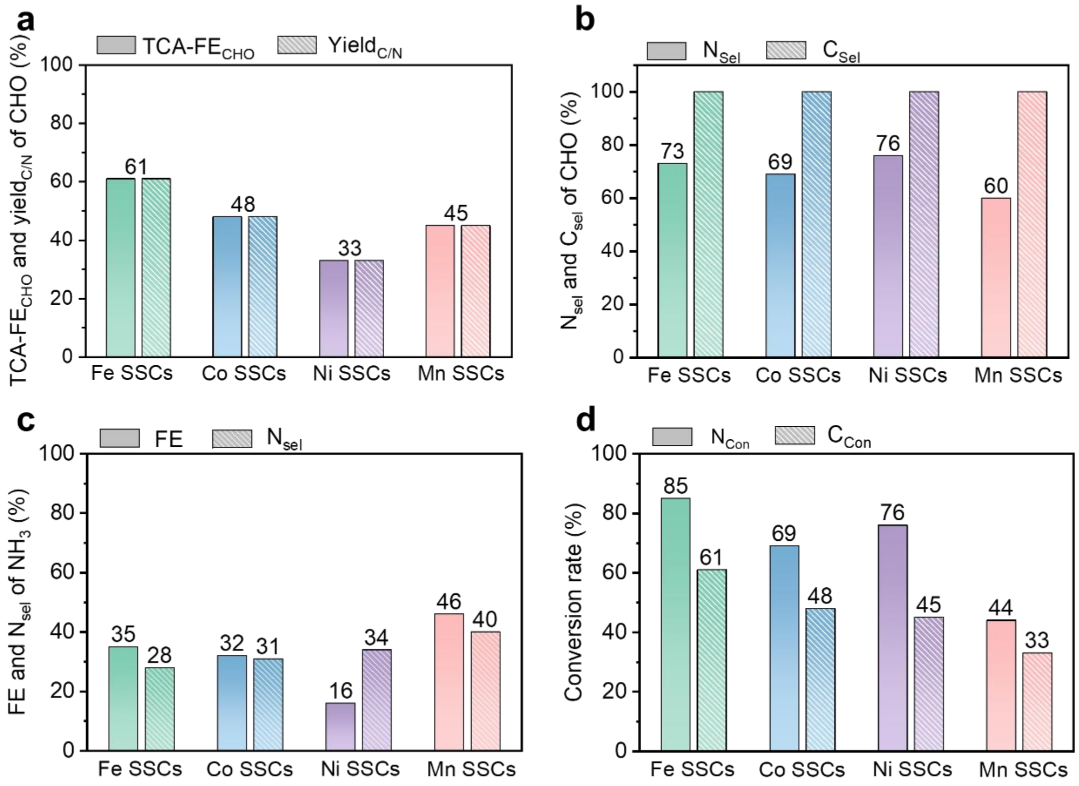

Figure S16. TCA-FE$_{CHO}$ & yield$_{C/N}$ of CHO **(a)**, N selectivity & C selectivity **(b)**, Faradaic efficiency & N selectivity of NH_3 **(c)** and N conversion rate & C conversion rate **(d)** of electrocatalytic synthesis of CHO over M SSCs electrocatalysts (M= Fe, Co, Ni, Mn). The constant electrolysis was all performed in 10 mL 0.5 M Na_2CO_3 with 0.5 M $NaNO_2$ and CYC at 20 mA cm^{-2} for 1930 C.

Although Ni SSCs show the highest selectivity of CHO and lowest selectivity of NH_3, the TCA-FE$_{CHO}$ and yield$_{C/N}$ of CHO are extremely low due to the good performance for HER. Therefore, based on comprehensive consideration of all indicators, Fe-D GSCs containing Fe-defect catalytic pairs may be an ideal catalyst for the near-stoichiometric conversion towards CHO at the high TCA-FE$_{CHO}$.

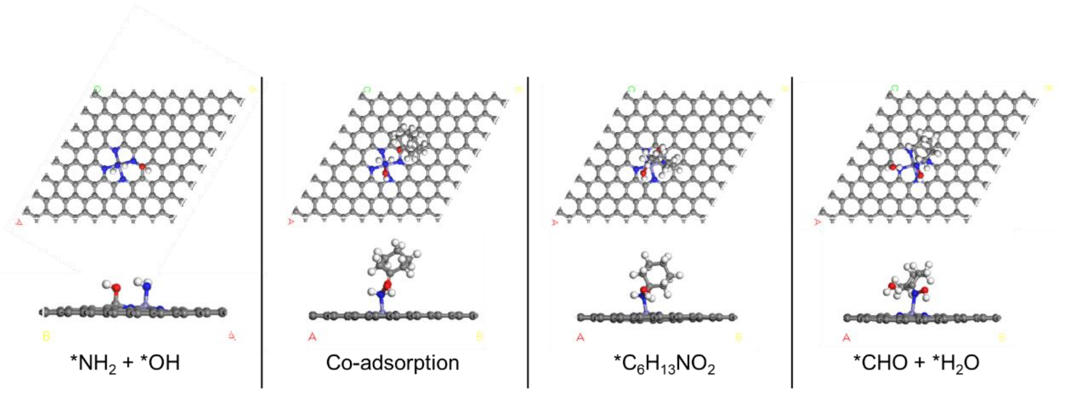

Figure S17. Structural models of the C-N coupling and over-reduction of *NH_2OH.

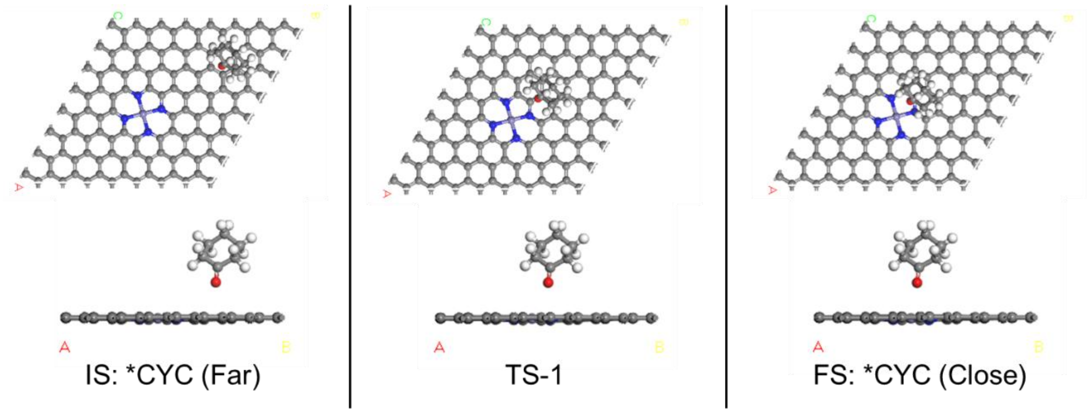

Figure S18. Structural models of the *CYC migration from the far site to the single Fe site.

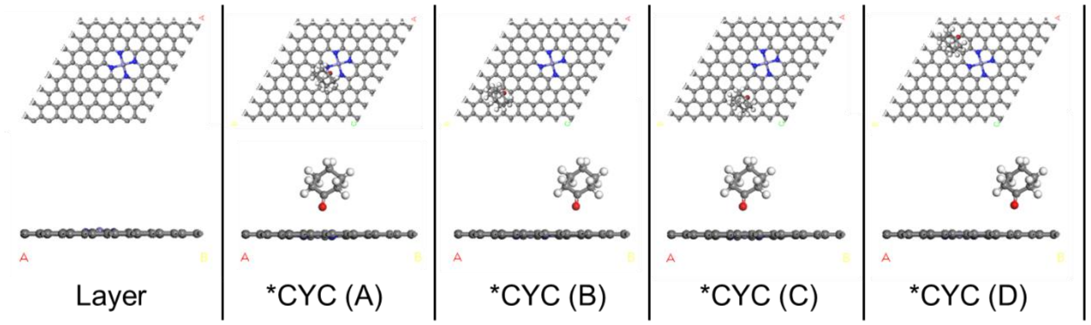

Figure S19. Structural models of *CYC adsorption over A site, B site, C site and D site over Fe SCCs electrocatalysts.

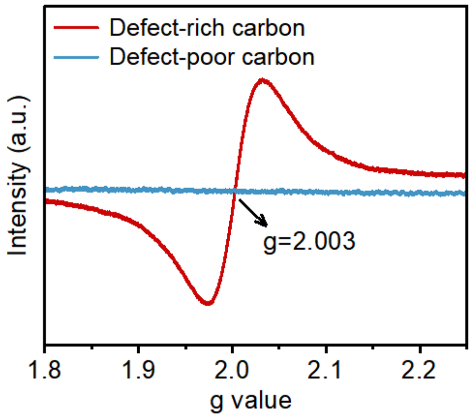

Figure S20. EPR spectra of defect-rich carbon (red line) and defect-poor carbon (blue line).

To investigate the effect of defect on CYC adsorption, purchased Vulcan XC72 was heated at 1000 ℃ for 2 hours with a heating rate of 5℃/min to obtain defect-poor carbon under Ar/H_2 atmosphere. Defect-rich carbon was synthesized based on as-synthesized defect-poor carbon via the argon plasma treatment. The sharp peak with the corresponding g = 2.003 in results of EPR spectra and the strong signal at 1320 cm^{-1} in Raman spectra both prove the high defect concentration construction of defect-rich carbon. In the further test of acetone-TPD tests, acetone is chosen as the probe molecule due to the similar structure of acetone and CYC.

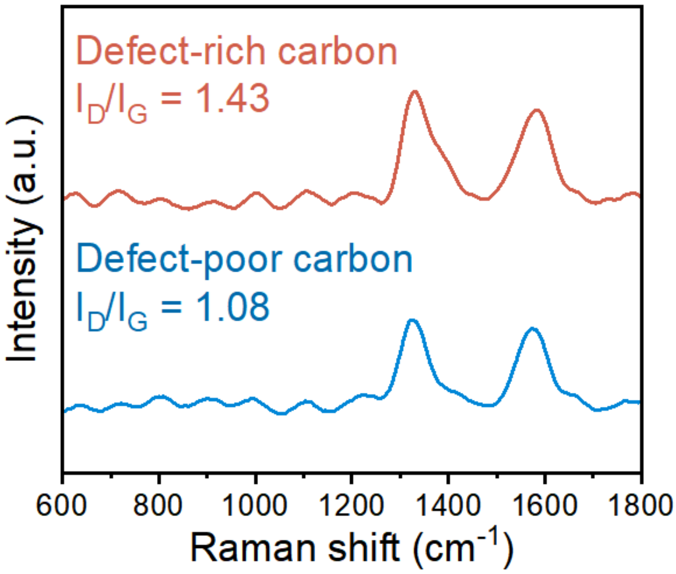

Figure S21. Raman spectra of the defect-poor carbon (blue line) and defect-rich carbon (red line).

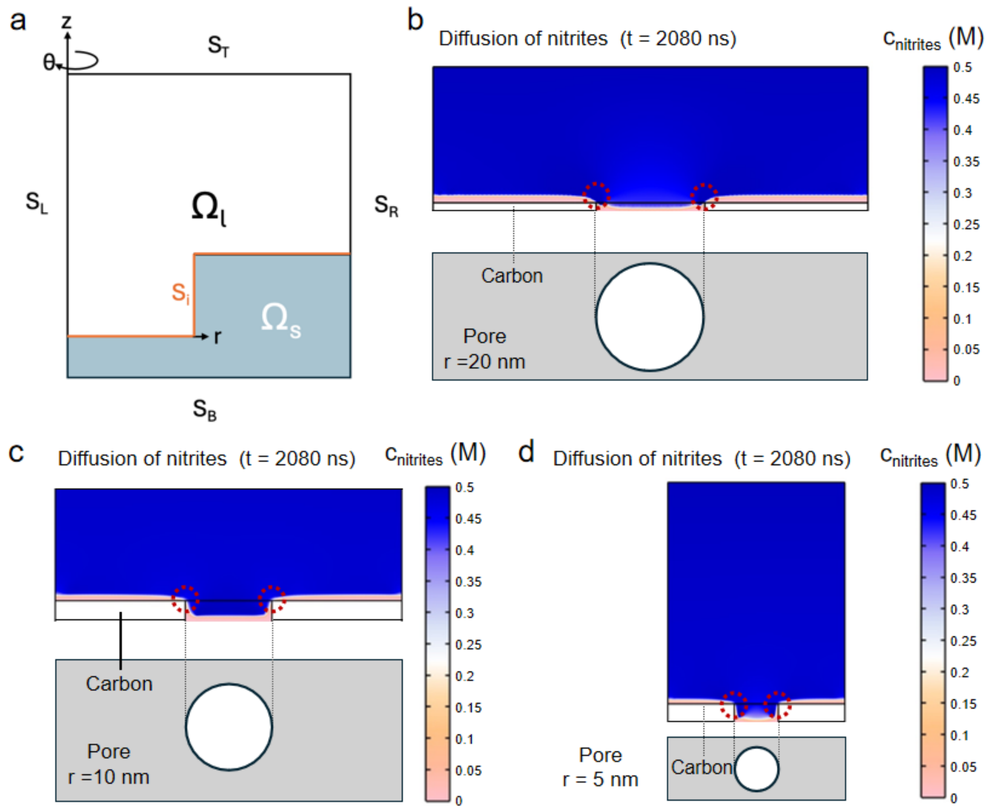

Figure S22. a) Schematic diagram of the simulation region in cylindrical coordinates, where Ω represents the bulk phase, S represents the surface. **b)** Surface NO_2^- density and distributions on the surface of carbon materials with 20 nm carbon defects. **c)** Surface NO_2^- density and distributions on the surface of carbon materials with 10 nm carbon defects. **d)** Surface NO_2^- density and distributions on the surface of carbon materials with 5 nm carbon defects.

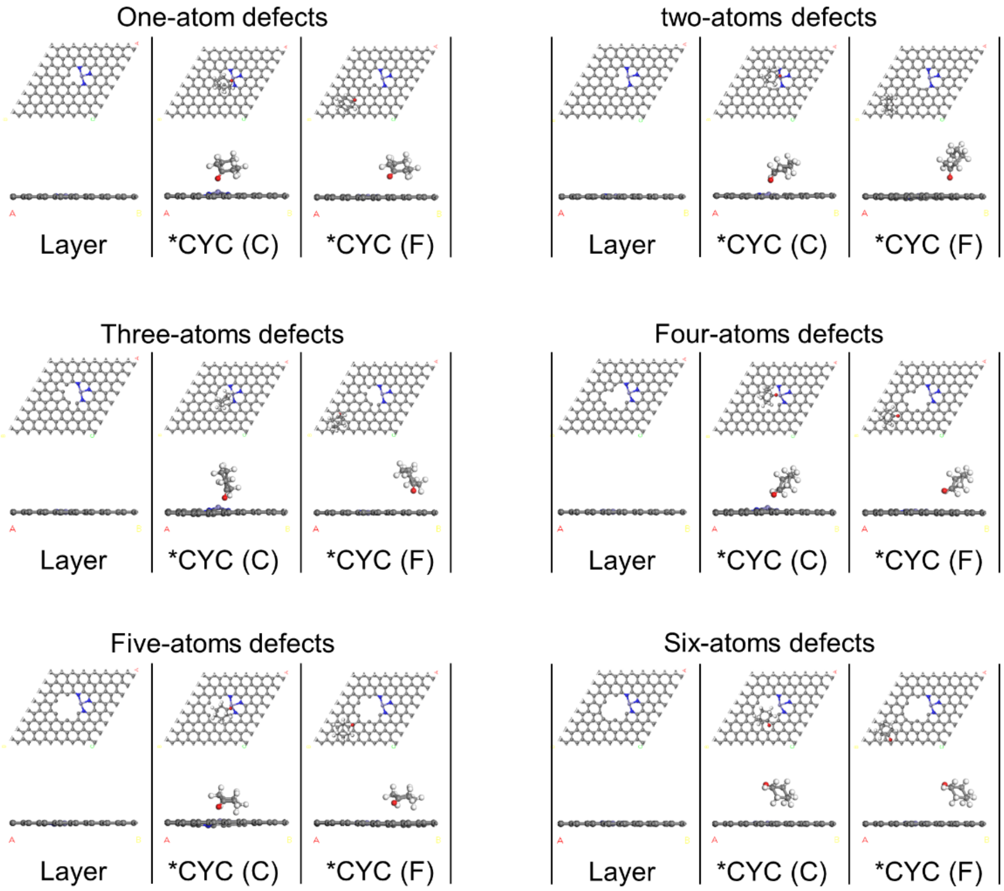

Figure S23. Structural models of *CYC adsorption on Fe-GDCs with defects of different sizes (ranging from one atom to six atoms).

According to the DFT results, the CYC adsorption at the Fe-defect catalytic pair always gives priority over that at the site far away from the isolated Fe site. Therefore, the Fe-defect catalytic pair can ensure the co-adsorption of CYC and NH_2OH for the subsequent C-N coupling towards CHO. Reasonably, the Fe-D GSCs without isolated Fe sites may be an ideal electrocatalyst for the near-stoichiometric conversion toward CHO

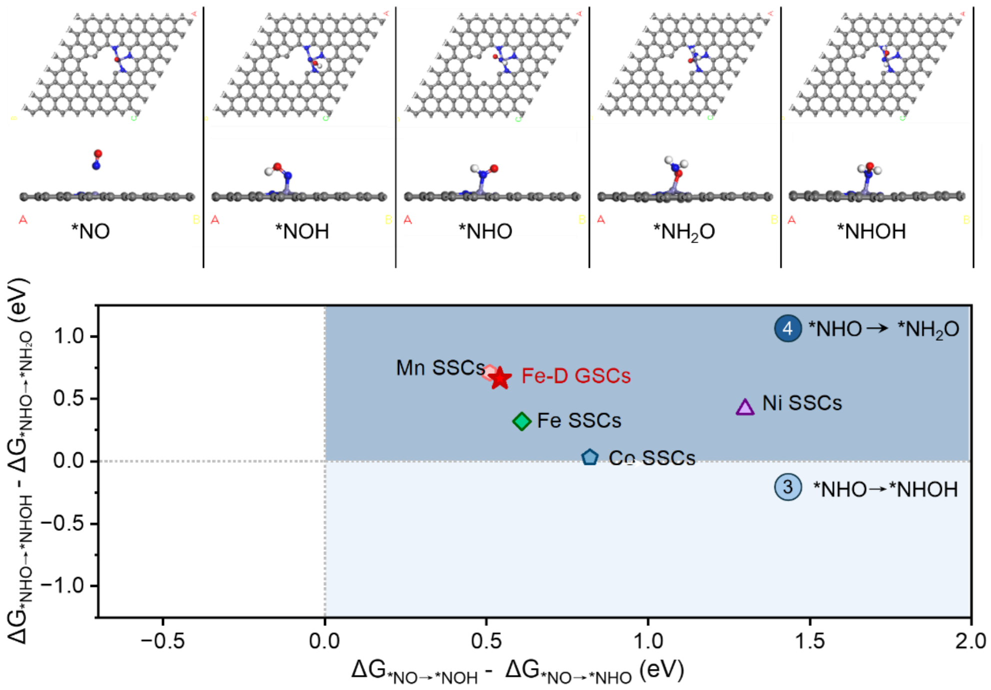

Figure S24. Comparison of the different NO_2RR pathways over Fe-D GSCs.

Based on the structure of the Fe-defect catalytic pair, the free energy changes (FEC) of various steps in NO_2RR were obtained via DFT calculations. The hydrogenation FEC of *NO to *NHO (-0.43 eV) is lower than that of *NO to *NOH (0.11 eV). Meanwhile, the hydrogenation FEC of *NHO to $*NH_2O$ (-1.09 eV) is lower than that of *NHO to *NHOH (-0.42 eV). Therefore, NO_2RR pathway over the Fe-defect pair follows the pathway involving $*NHO \rightarrow *NH_2O \rightarrow *NH_2OH$ (Pathway 4→8), thus favoring the formation of $*NH_2OH$ thermodynamically.

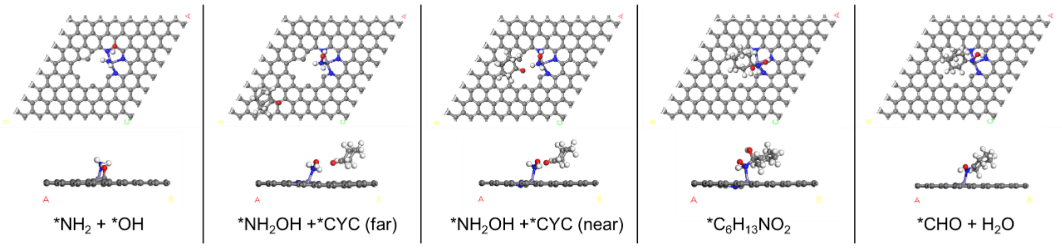

Figure S25. Structural models of the reaction pathways for the formed *NH$_2$OH at Fe-D$_4$ CP.

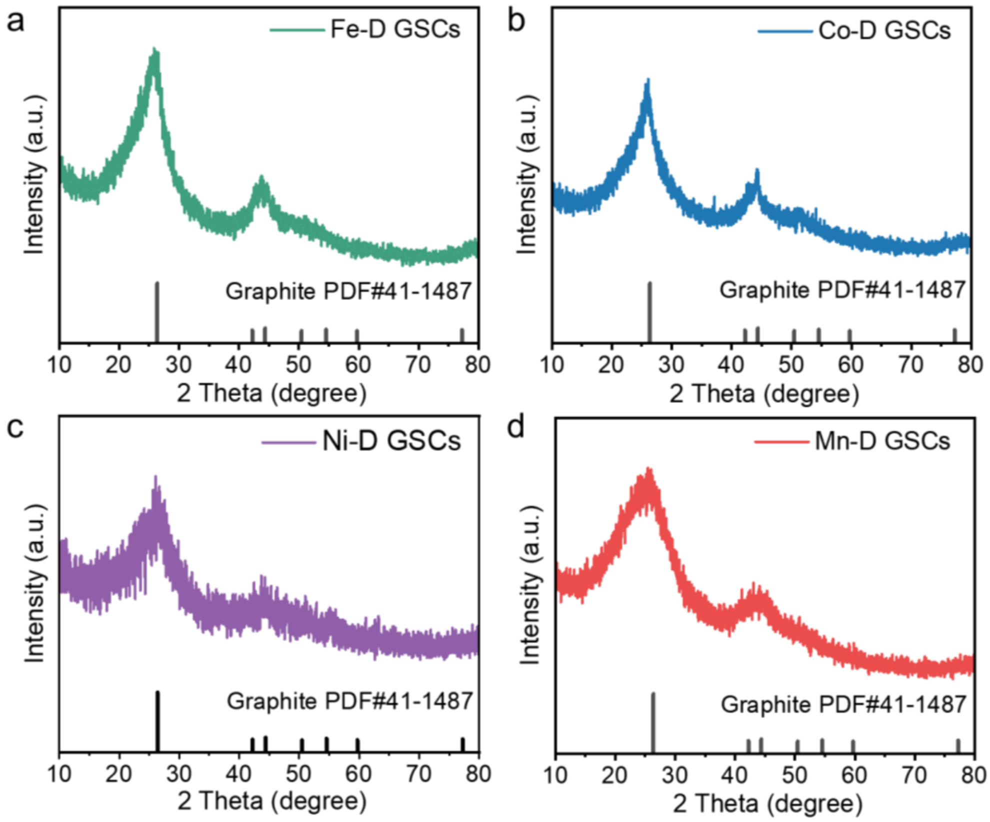

Figure S26. XRD patterns for Fe-D GSCs **(a)**, Co-D GSCs **(b)**, Ni-D GSCs **(c)** and Mn-D GSCs **(d)**.

There are no obvious signals of metal nanoparticles or metal oxide nanoparticles on series of M-D SSCs.

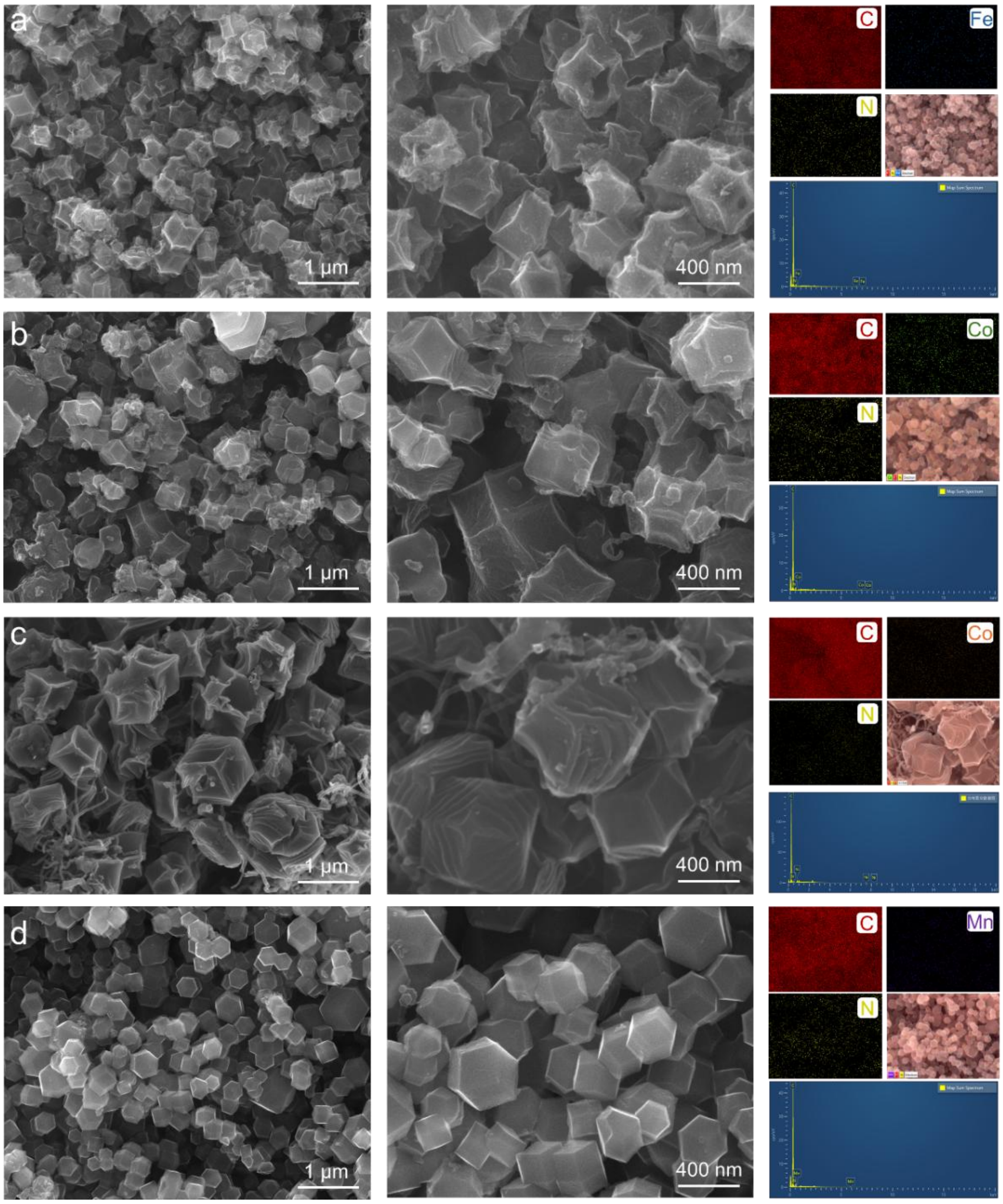

Figure S27. SEM images for Fe-D GSCs **(a)**, Co-D GSCs **(b)**, Ni-D GSCs **(c)** and Mn-D GSCs **(d)**.

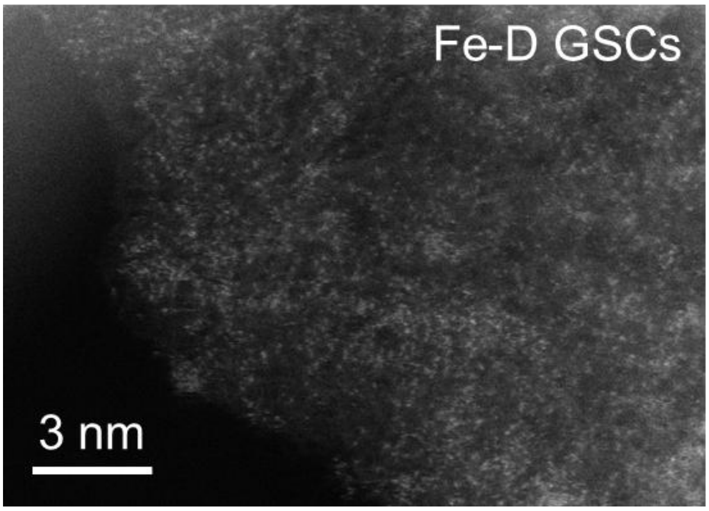

Figure S28. HAADF-STEM image of Fe-D GSCs.

The Fe-D GSCs were synthesized via the pyrolysis of ZIF-8 absorbing ferrous-citrate complexes. The coordination interaction between Zn ions in ZIF-8 and citrates in ferrous-citrate complexes can maximum the numbers of active sites after pyrolysis. The electrolysis experiments all use the Fe-D GSCs because of more Fe-D catalytic pairs in this electrocatalyst.

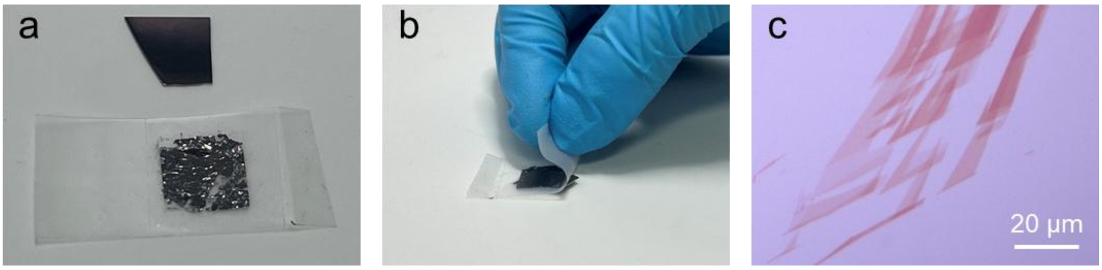

Figure S29. a, Optical image of the SiO_2/Si substrate and adhesive tape with graphite flakes, which adhere after contact with a HOPG crystal. **b,** Removal of the substrate from the hot plate and peeling off of the tape. **c**, Optical micrograph of one of the graphene flaks.

The structure can't be observed due to the 3D-structure of Fe-D GSCs or Fe SSCs supported on ZIF8-derived N-doped carbon. Therefore, to acquire image of high quality for observing Fe-defect catalytic pair, NH_3-treated few-layer graphene (FG) was used as the carbon support.

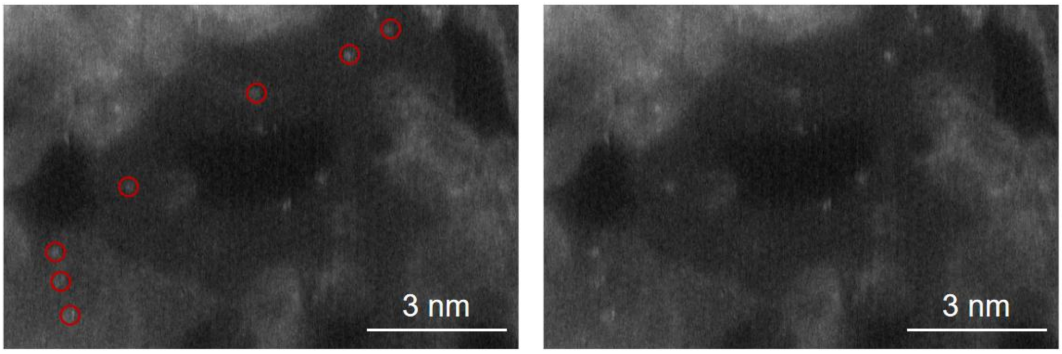

Figure S30. HAADF-STEM images of Fe SSCs/FG.

Firstly, multiple-layer graphene (FG) was calcined under NH_3 atmosphere for 2 hours at 400°C (a heating rate of 5°C min^{-1}). Then FG was spin coated with 50 μl 1000 ppb iron phthalocyanine solution. After calcining at 600°C for two hours, the Fe SSCs/FG were obtained. There are a few defects existing around the single Fe sites for Fe SSCs/FG, and numerous single Fe sites are loaded on the non-defect layer of graphene. The reason why the single Fe site can still locate at the edge sites is the lower formation energy of Fe-defect catalytic pair.

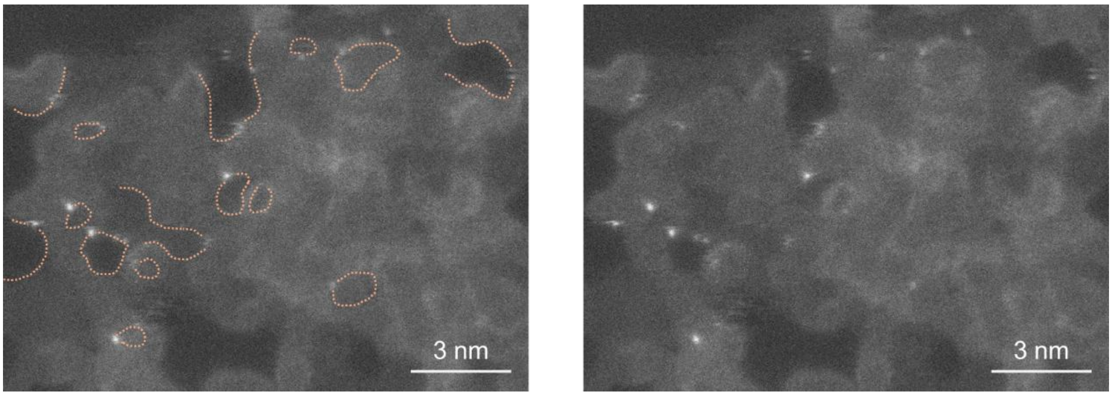

Figure S31. HAADF-STEM images of Fe-D GSCs/FG.

Firstly, FG was calcined under NH_3 atmosphere for 2 hours at 400°C (a heating rate of 5°C min^{-1}). Then FG was spin coated with 50 μl 1000 ppb ferrous-citrate solution. After calcining at 600°C for two hours, the Fe-D GSCs/FG were obtained. As shown in Figure S31, almost every isolated Fe site borders on the defect sites on the Fe-D GSCs/FG.

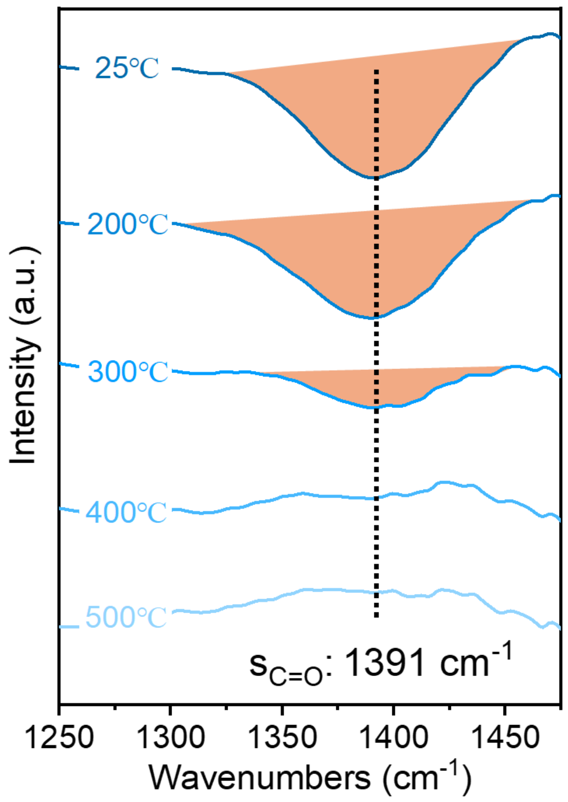

Figure S32. FTIR spectra of different temperatures during the pyrolysis process of Fe-CA/NC.

Fe-CA/NC is the ZIF8-derived N-doped carbon material absorbing ferrous-citrate complexes. It is well known that the pyrolysis of ZIF8 will release the volatile component. To avoid the interference signal resulting from the pyrolysis of ZIF8, we used Fe-CA/NC as precursors in this experiment and TG-MS tests.

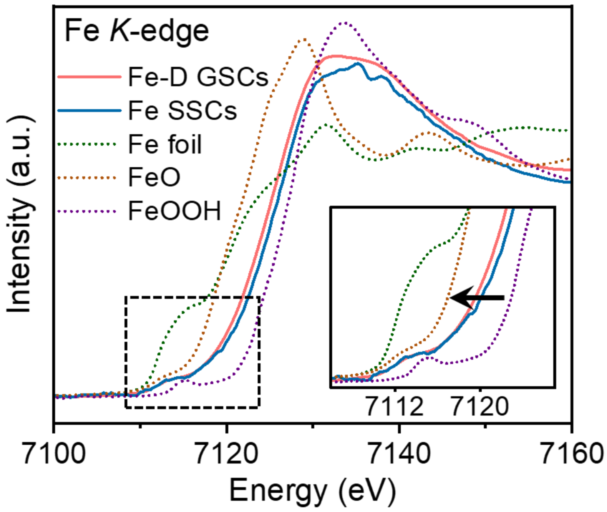

Figure S33. Normalized Fe K-edge XANES spectra of Fe SSCs (blue line), Fe-D GSCs (red line), and reference samples (e.g., Fe-foil, FeO, and FeOOH). The inset shows a magnified view of the pre-edge peaks.

X-ray absorption near-edge structure spectra of the Fe K edge confirmed that the Fe oxidation state in Fe SSCs was higher than that in Fe-D GSCs. This phenomenon should be resulted from the existence of accompanying defects close to isolated Fe sites.

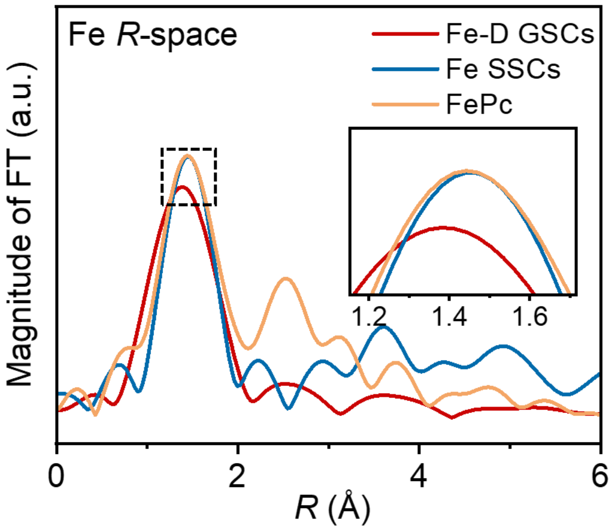

Figure S34. Fourier-transformed EXAFS in real-space (R-space) of Fe SSCs, Fe-D GSCs and Fe phthalocyanine (FePc) standard sample.

The intensity of Fe SSCs is almost the same as that of Fe phthalocyanine (FePc), indicating that the coordination number (CN) of Fe SSCs is also similar as that of Fe phthalocyanine. Similarly, the CN of Fe-D GSCs for Fe-N/O paths should be lower than that of Fe SSCs. This phenomenon might be caused by the existence of accompanying nitrogen defects near isolated Fe sites.

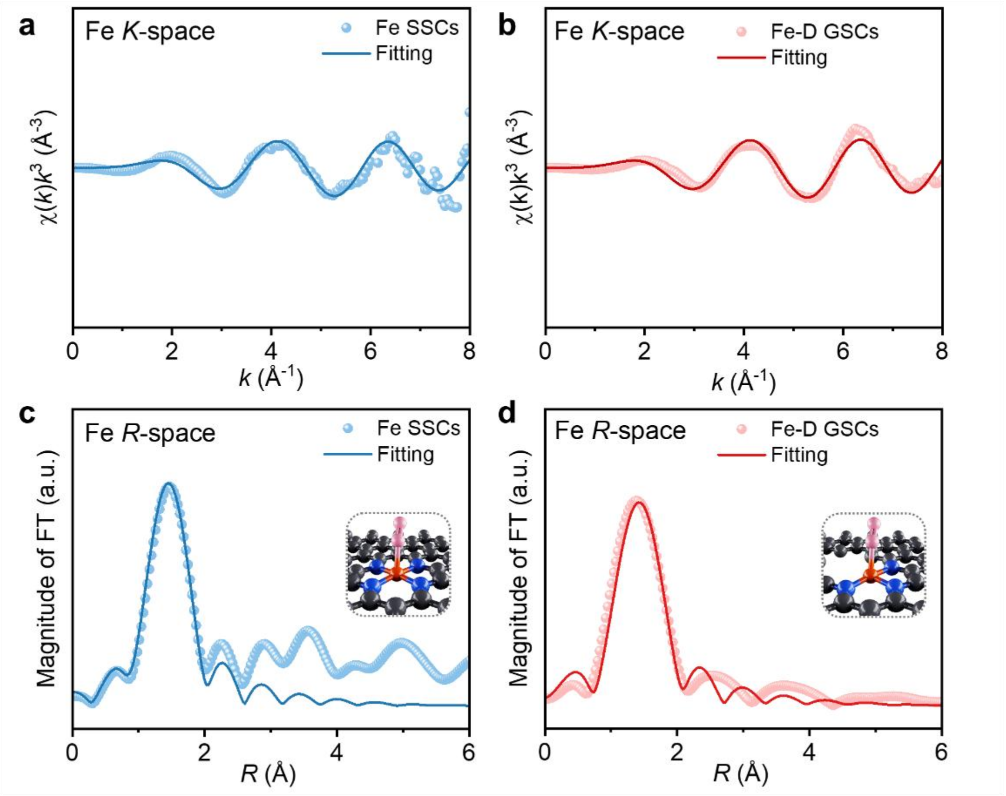

Figure S35. Fitting curves of the EXAFS of Fe SSCs (**a**, blue line) and Fe-D GSCs (**b**, red line) in the *k*-space. Fitting curves of the EXAFS of Fe SSCs (**c**, blue line) and Fe-D GSCs (**d**, red line) in the *R*-space.

The coordination numbers of the N and O atoms in the first coordination sphere of Fe-D GSCs are estimated to be 2.8 and 1.0 at distances of 1.92 and 2.04 Å, respectively (Table S9). Meanwhile, the coordination numbers of the N and O atoms in the first coordination sphere of Fe SSCs are estimated to be 3.7 and 0.8 at distances of 1.92 and 2.04 Å, respectively (Table S9). Therefore, the coordination numbers for Fe-N paths in Fe-D GSCs (CN: 2.8) are nearly one coordination number less than that in Fe SSCs (CN: 3.7), indicating that there was almost an accompanying N defect near each isolated Fe site in Fe-D GSCs.

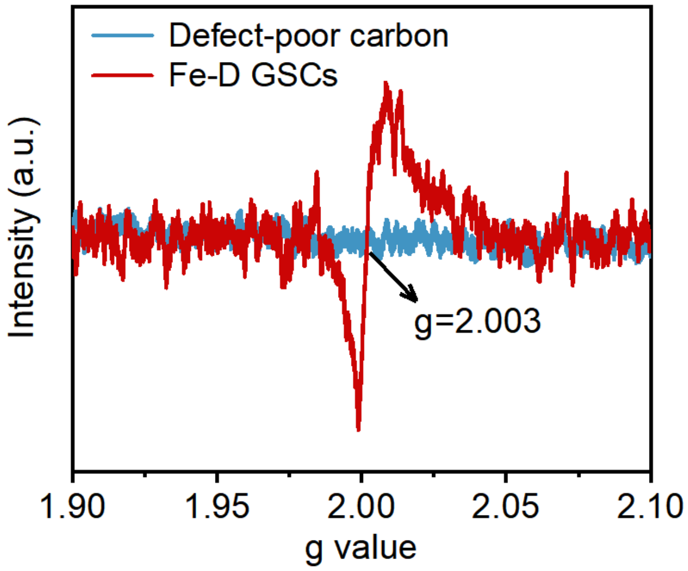

Figure S36. EPR spectra of defect-poor carbon (blue line) and Fe-D GSCs (red line).

Ferromagnetism in Fe-D GSCs will largely decrease the signal of carbon sites with unpaired electrons. Meanwhile, intrinsic defects of carbon will affect the investigation about the pyrolysis of iron-citrate complex. Therefore, as-synthesized defect-poor carbon was used to synthesize Fe-D GSCs to investigate the pyrolysis of ferrous-citrate complexes. The sharper peak with the corresponding g = 2.003 after pyrolysis reflects the existence of carbon sites with unpaired electron, thus proving the appearance of accompanying defects.

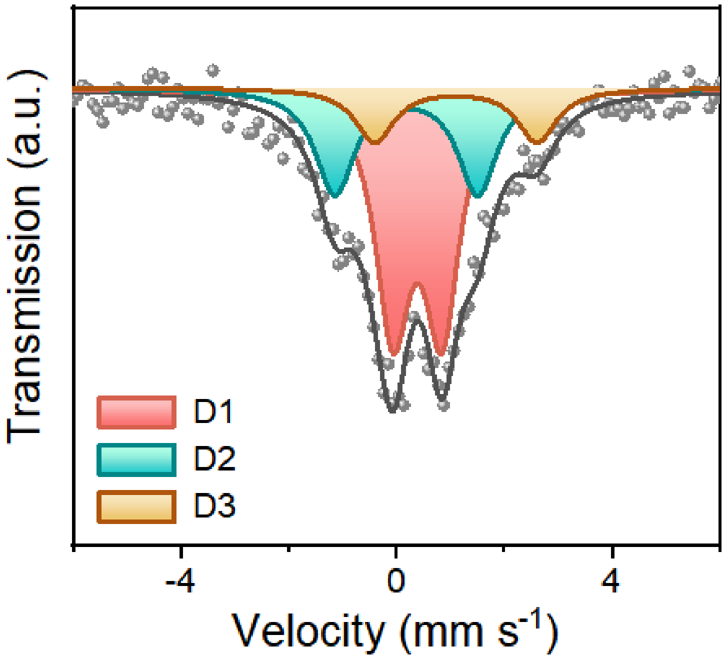

Figure S37. Fe Mössbauer spectra of the Fe-D GSCs at room temperatures.

Fe Mössbauer spectra, which are highly sensitive to electron distribution and coordination environment of Fe atoms, were acquired to investigate electron distribution of Fe atoms in the Fe-defect catalyst. Mossbauer spectrum of the Fe-defect catalyst can be well fitted using three quadrupole doublets (isomer shift (IS) = 0.39 mm s^{-1}, quadrupole splitting (QS) = 0.91 mm s^{-1}(D1), IS = 0.18 mm s^{-1}, QS = 2.64 mm s^{-1}(D2), IS = 1.10 mm s^{-1}, QS = 3.01 mm s^{-1}(D3)), which can generally be attributed to high spin Fe (III), low spin Fe (II) and high spin Fe (II) species. IS values represent the electron density of Fe atoms, and QS values increase with the asymmetry in electron distribution. The QS values of D1 (0.91 mm s^{-1}) in the Fe-defect catalyst is obviously increased compared with that (0.7 mm s^{-1}) in previous literature of Fe-N_4 structure, reflecting the electron distribution of Fe atoms of D1 is likely to be affected by accompanying defects. Meanwhile, the high QS values of D2 and D3 also imply the increasing asymmetry around the Fe atoms in the Fe-defect catalyst, which is also caused by the formation of accompanying defects. The fitting results for the Mossbauer spectrum of the Fe-defect catalyst generally but ambiguously proved that almost each isolated Fe site in the Fe-defect catalyst matched with an accompanying defect to form a Fe-defect catalytic pair. Therefore, the Fe-defect catalyst without isolated Fe sites is a promising electrocatalysts for near-stoichiometric conversion towards CHO at complete conversion and high FE.

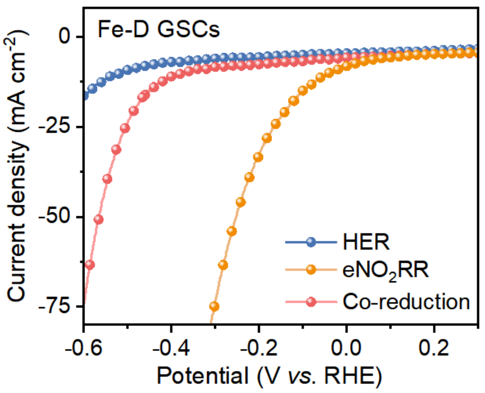

Figure S38. Electrocatalytic performance of HER (blue line), eNO_2RR (orange line) and electrocatalytic synthesis of CHO (red line) over Fe-D GSCs at a scan rate of 5 mV/s. The electrolyte of these tests is 0.5 M Na_2CO_3.

After adding 0.5 M CYC into the 0.5 M Na_2CO_3 with 0.5 M $NaNO_2$, the current density largely decreased both on the Fe-D GSCs and Fe SSCs. This phenomenon might be caused by the strong adsorption of CYC on carbon materials.

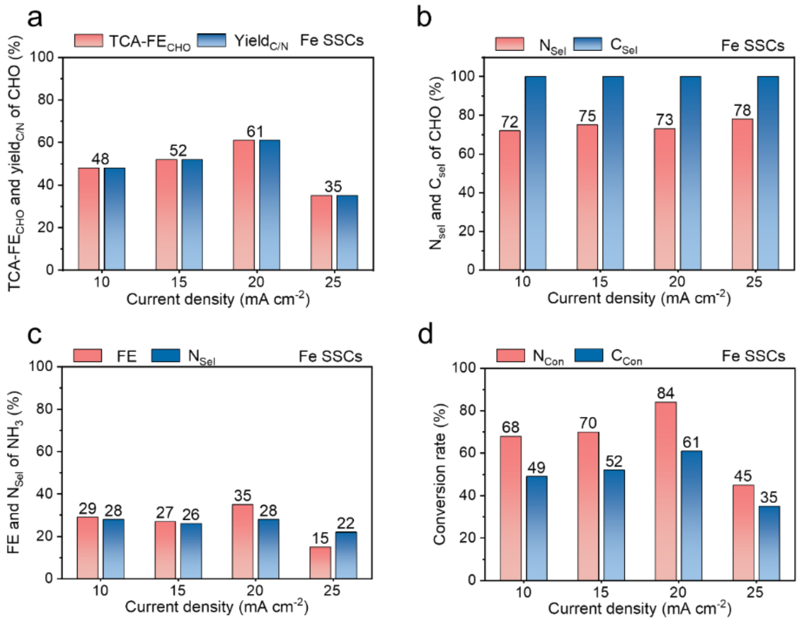

Figure S39. Indicators of the electrocatalytic synthesis of CHO over Fe SSCs at different current density for 1930 C. **(a)** TCA-FE_{CHO} and $yield_{C/N}$ of CHO. **(b)** N_{sel} and C_{sel} of CHO. **(c)** Faradaic efficiency and N_{sel} of NH_3. **(d)** Conversion rate of $NaNO_2$ (N_{Con}) and and conversion rate of CYC (C_{Con}).

The best performance for the electrocatalytic synthesis of CHO on Fe SSCs was obtained at a current density of 20 mA cm^{-2}. The TCA-FE_{CHO}, $yield_{C/N}$, N selectivity and C selectivity of CHO on Fe SSCs reached 61.2%, 61.2%, 72.5% and ~100%, respectively.

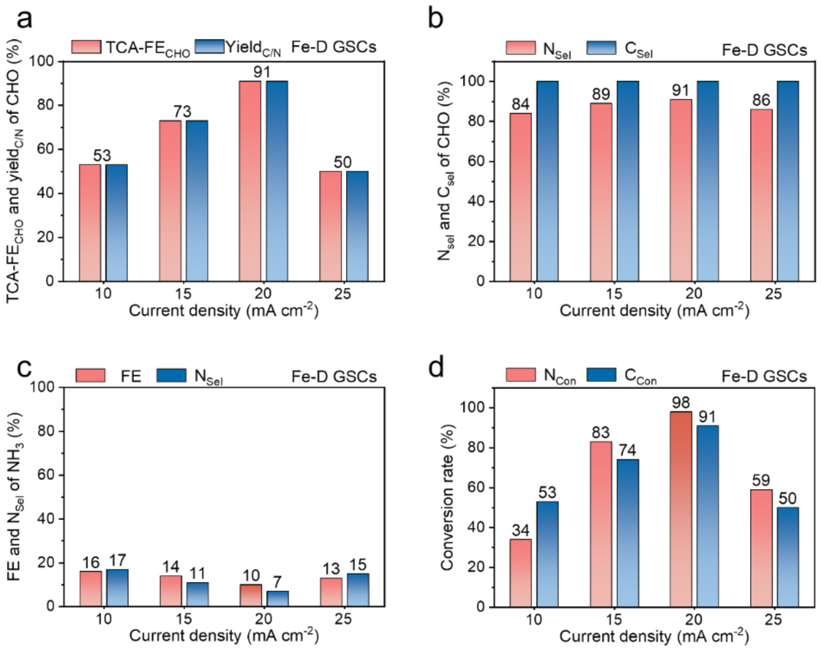

Figure S40. Indicators of the electrocatalytic synthesis of CHO over Fe-D GSCs at different current density for 1930 C. **(a)** TCA-FE_{CHO} and $yield_{C/N}$ of CHO. **(b)** N_{sel} and C_{sel} of CHO. **(c)** Faradaic efficiency and N_{sel} of NH_3. **(d)** Conversion rate of $NaNO_2$ (N_{Con}) and conversion rate of CYC (C_{Con}).

The best performance for the electrocatalytic synthesis of CHO on Fe-D GSCs was also obtained at a current density of 20 mA cm^{-2}. The TCA-FE_{CHO}, $yield_{C/N}$, N selectivity and C selectivity of CHO on Fe-D GSCs reached 61.2%, 61.2%, 72.5% and ~100%, respectively.

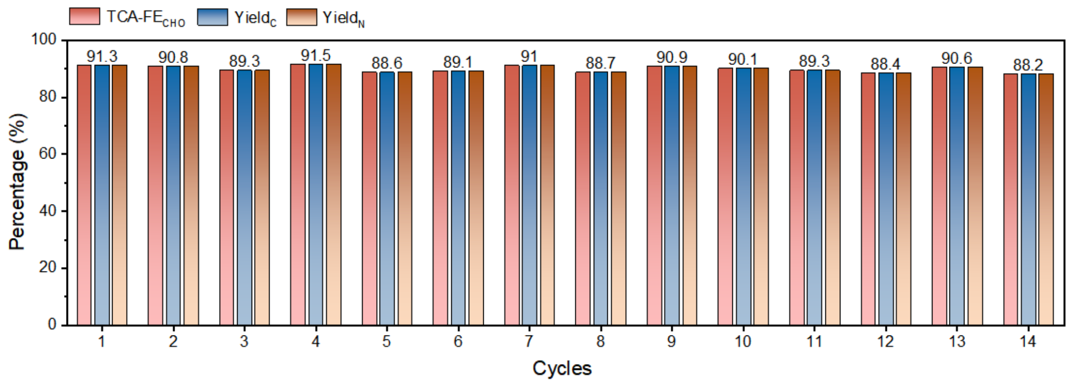

Figure S41. Stability measurement of Fe-D GSCs for 14 cycles.

The C yield, N yield and TCA-FE_{CHO} remained above 88 %, respectively, for 14 cycles in a 10 mL 0.5 M Na_2CO_3 electrolyte with 0.5 M CYC and 0.5 M $NaNO_2$ at a current density of 20 mA cm^{-2}, demonstrating the excellent stability of Fe-D GSCs. Therefore, for the electrocatalytic synthesis of CHO on Fe-D GSCs, various indicators at high complete conversion satisfies the demands of industrialization, and unit costs of raw material costs are largely decreased.

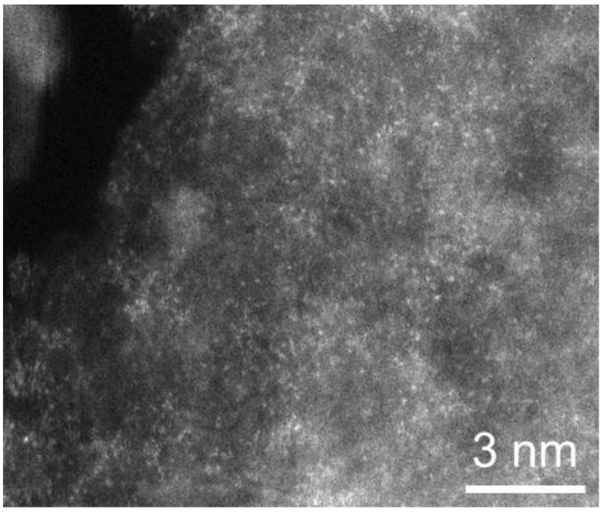

Figure S42. AC-STEM images for Fe-D GSCs after reaction. This image proves that there are no obvious metal clusters or nanoparticles.

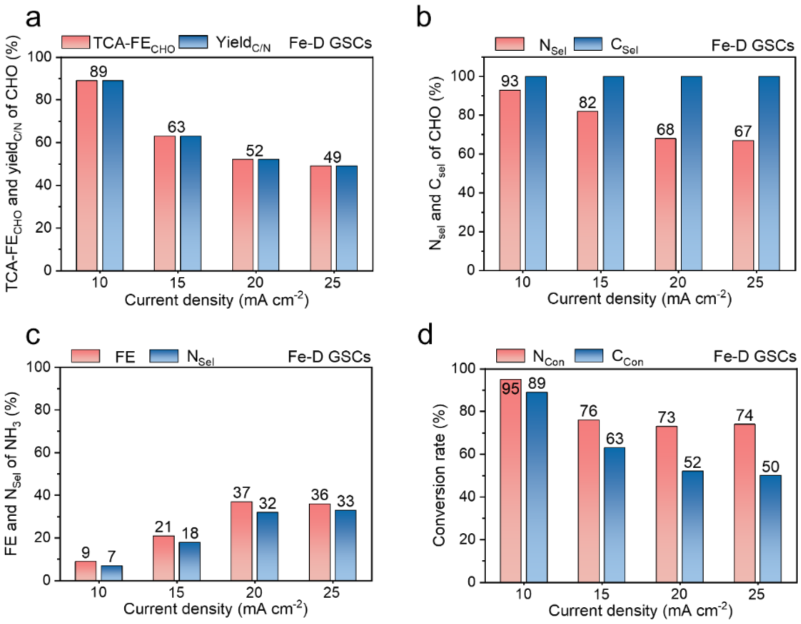

Figure S43. Indicators of this oxime-alkali process over Fe-D GSCs at different current density for 1930 C in 0.5 M KNO_2 with 0.5 M CYC. **(a)** TCA-FE_{CHO} and yield$_{C/N}$ of CHO. **(b)** N_{sel} and C_{sel} of CHO. **(c)** Faradaic efficiency and N_{sel} of NH_3. **(d)** Conversion rate of $NaNO_2$ (N_{Con}) and conversion rate of CYC (C_{Con}).

The best performance for this oxime-alkali process on Fe-D GSCs was also obtained at a current density of 10 mA cm^{-2}. The TCA-FE_{CHO}, yield$_{C/N}$, N selectivity and C selectivity of CHO on Fe-D GSCs can still reach 89%, 89%, 93% and ~100%, respectively. In the absence of additional electrolyte (0.5 M Na_2CO_3), the optimal reaction current density decreases from 20 mA cm^{-2} to 10 mA cm^{-2} due to the increase of the liquid resistance in the H-type cell.

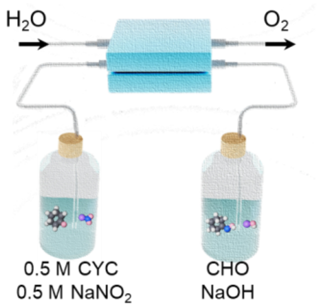

Cathode reaction: $NO_2^- + C_6H_{10}O + 3H_2O - 4e^- = C_6H_{11}NO + 5OH^-$

Anode reaction: $4OH^- + 4e^- = 2H_2O + O_2$

Total reaction (NO_2^-): $NO_2^- + C_6H_{10}O + H_2O = C_6H_{11}NO + OH^- + O_2$

Figure S44. Schematic diagrams of the cathodic oxime-alkali process.

In this reaction, given the relatively high concentration of the substrate (0.5 M $NaNO_2$), it is sufficient to be directly used as the electrolyte. The cathodic and anodic reaction equations are shown in Figure S44. At the cathode, C–N coupling occurs to form oximes, while the oxygen evolution reaction (OER) takes place at the anode. The overall reaction equation indicates that hydroxide ions are still generated at the cathode, resulting in the formation of a NaOH solution.

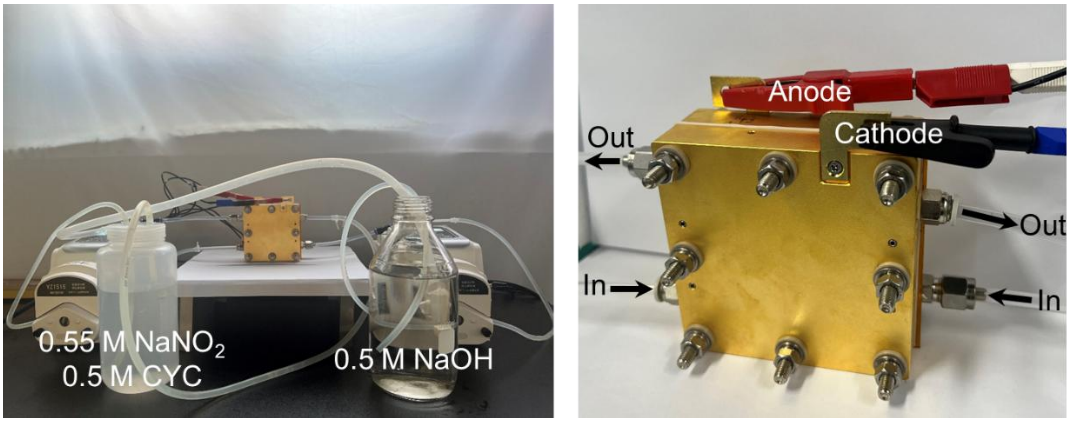

Figure S45. Photos for the 5 cm × 5 cm flow cell.

In the test of H-type cell, 0.5 M $NaNO_2$ and 0.5 CYC can't be thoroughly converted to CHO and NaOH. The rest of CHO will increase the separation cost of CHO so that the feedings are changed to 0.55 M $NaNO_2$ and 0.5 M CYC to ensure the complete conversion of CYC. Therefore, the mixture after reaction consists of NaOH, NH_3 and CHO. Firstly, the CHO is extracted from the mixture using ethyl acetate. And then the NH_3 can be removed from the mixture via heating. Finally, high concentration NaOH solution can be obtained.

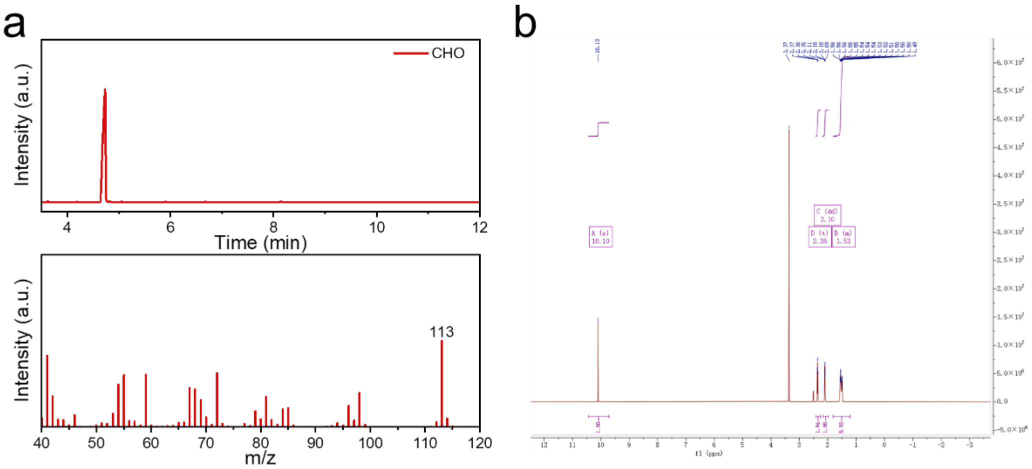

Figure S46. a, GC-spectrum and MS-spectrum of electrocatalyticly synthesized CHO over Fe-D GSCs in the flow reactor. **b,** 1H NMR spectrum of electrocatalyticly synthesized CHO over Fe-D GSCs in the flow reactor.

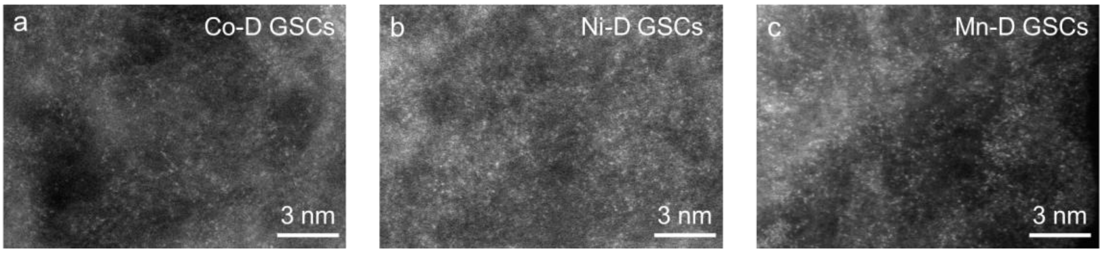

Figure S47. AC-STEM images for Co-D GSCs **(a)**, Ni-D GSCs **(b)** and Mn-D GSCs **(c)**. These images prove that there are no obvious metal clusters or nanoparticles.

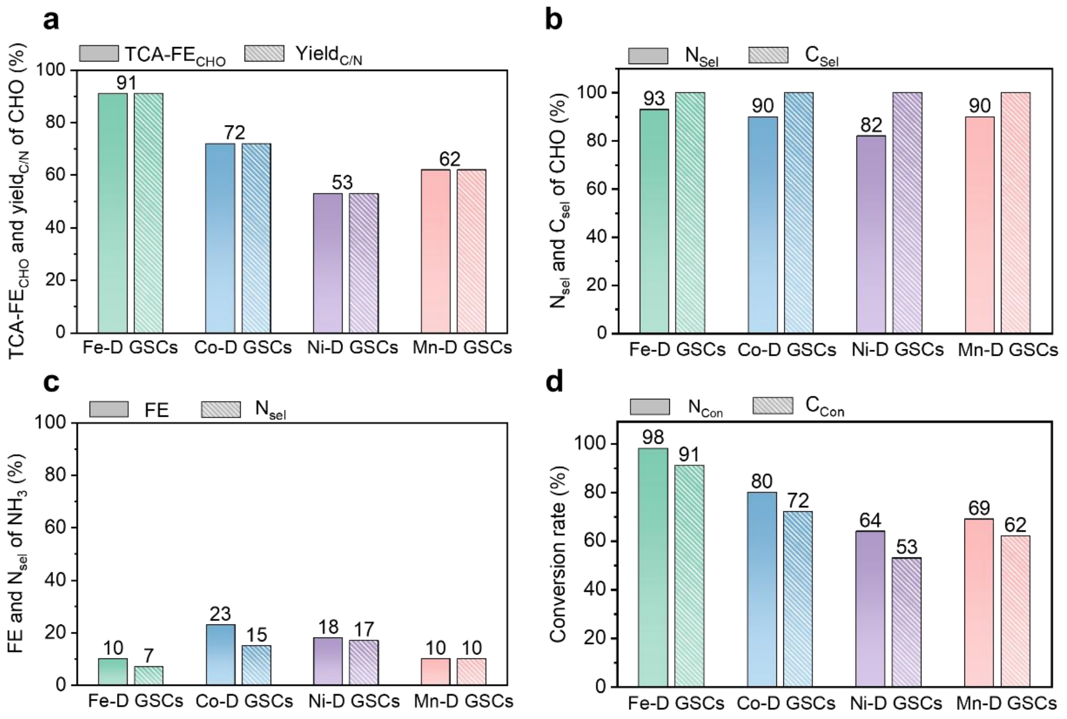

Figure S48. Indicators of the electrocatalytic synthesis of CHO over series M-D GSCs at different current density for 1930 C. **(a)** TCA-FE$_{CHO}$ and yield$_{C/N}$ of CHO. **(b)** N_{sel} and C_{sel} of CHO. **(c)** Faradaic efficiency and N_{sel} of NH_3. **(d)** Conversion rate of $NaNO_2$ (N_{Con}) and conversion rate of CYC (C_{Con}).

SUPPLEMENTARY TABLES

Table S1. Calculated values for free energy, and free energy change of NO_2RR over Fe (110), Co (111) and Ni (111).

Intermediates	G-Fe (110)	ΔG	Fe (110)
*NO	G_0 = -579.97 eV		
*NOH	G_{1-1} = -582.93 eV	$\Delta G_{0\rightarrow 1-1} = G_{1-1} - G_0 - 1/2G_{H2}$	0.45 eV
*NHO	G_{1-2} = -582.63 eV	$\Delta G_{0\rightarrow 1-2} = G_{1-2} - G_0 - 1/2G_{H2}$	0.75 eV
*N	G_{2-1} = -572.47 eV	$\Delta G_{1-1\rightarrow 2-1} = G_{2-1} - G_{1-1} - 1/2G_{H2} + G_{H2O}$	-0.27 eV
*NHOH	G_{2-2} = -586.08 eV	$\Delta G_{1-1\rightarrow 2-2} = G_{2-2} - G_{1-1} - 1/2G_{H2}$	0.25 eV
**Intermediates**	**G-Co (111)**	**ΔG**	**Co (111)**
*NO	G_0 = -340.58 eV		
*NOH	G_{1-1} = -343.15 eV	$\Delta G_{0\rightarrow 1-1} = G_{1-1} - G_0 - 1/2G_{H2}$	0.83 eV
*NHO	G_{1-2} = -343.00 eV	$\Delta G_{0\rightarrow 1-2} = G_{1-2} - G_0 - 1/2G_{H2}$	0.98 eV
*N	G_{2-1} = -333.77 eV	$\Delta G_{1-1\rightarrow 2-1} = G_{2-1} - G_{1-1} - 1/2G_{H2} + G_{H2O}$	-1.35 eV
*NHOH	G_{2-2} = -347.05 eV	$\Delta G_{1-1\rightarrow 2-2} = G_{2-2} - G_{1-1} - 1/2G_{H2}$	-0.50 eV
**Intermediates**	**G-Ni (111)**	**ΔG**	**Ni (111)**
*NO	G_0 = -256.48 eV		
*NOH	G_{1-1} = -259.43 eV	$\Delta G_{0\rightarrow 1-1} = G_{1-1} - G_0 - 1/2G_{H2}$	0.45 eV
*NHO	G_{1-2} = -258.90 eV	$\Delta G_{0\rightarrow 1-2} = G_{1-2} - G_0 - 1/2G_{H2}$	0.99 eV
*N	G_{2-1} = -250.25 eV	$\Delta G_{1-1\rightarrow 2-1} = G_{2-1} - G_{1-1} - 1/2G_{H2} + G_{H2O}$	-1.55 eV
*NHOH	G_{2-2} = -262.55 eV	$\Delta G_{1-1\rightarrow 2-2} = G_{2-2} - G_{1-1} - 1/2G_{H2}$	0.29 eV

Table S2. Calculated values for free energy, and free energy change of NO_2RR over Fe SSCs, Co SSCs, Ni SSCs and Mn SSCs.

Intermediates	G-Fe SSCs	ΔG	Fe SSCs
*NO	G_0 = -1181.82 eV		
*NOH	G_{1-1} = -1184.09 eV	$\Delta G_{0\rightarrow 1-1} = G_{1-1} - G_0 - 1/2G_{H2}$	1.13 eV
*NHO	G_{1-2} = -1184.69 eV	$\Delta G_{0\rightarrow 1-2} = G_{1-2} - G_0 - 1/2G_{H2}$	0.53 eV
*NHOH	G_{2-2} = -1188.17 eV	$\Delta G_{1-2\rightarrow 2-2} = G_{2-2} - G_{1-2} - 1/2G_{H2}$	-0.07 eV
*NH_2O	G_{2-3} = -1188.49 eV	$\Delta G_{1-2\rightarrow 2-3} = G_{2-3} - G_{1-2} - 1/2G_{H2}$	0.39 eV
**Intermediates**	**G-Co SSCs**	**ΔG**	**Co SSCs**
*NO	G_0 = -1180.25 eV		
*NOH	G_{1-1} = -1182.49 eV	$\Delta G_{0\rightarrow 1-1} = G_{1-1} - G_0 - 1/2G_{H2}$	1.16 eV
*NHO	G_{1-2} = -1183.31 eV	$\Delta G_{0\rightarrow 1-2} = G_{1-2} - G_0 - 1/2G_{H2}$	0.34 eV
*NHOH	G_{2-2} = -1186.83 eV	$\Delta G_{1-2\rightarrow 2-2} = G_{2-2} - G_{1-2} - 1/2G_{H2}$	-0.12 eV
*NH_2O	G_{2-3} = -1186.86 eV	$\Delta G_{1-2\rightarrow 2-3} = G_{2-3} - G_{1-2} - 1/2G_{H2}$	-0.15 eV
**Intermediates**	**G-Ni SSCs**	**ΔG**	**Ni SSCs**
*NO	G_0 = -1178.88 eV		
*NOH	G_{1-1} = -1180.80 eV	$\Delta G_{0\rightarrow 1-1} = G_{1-1} - G_0 - 1/2G_{H2}$	1.48 eV
*NHO	G_{1-2} = -1182.10 eV	$\Delta G_{0\rightarrow 1-2} = G_{1-2} - G_0 - 1/2G_{H2}$	0.18 eV
*NHOH	G_{2-2} = -1185.53 eV	$\Delta G_{1-2\rightarrow 2-2} = G_{2-2} - G_{1-2} - 1/2G_{H2}$	-0.03 eV
*NH_2O	G_{2-3} = -1185.95 eV	$\Delta G_{1-2\rightarrow 2-3} = G_{2-3} - G_{1-2} - 1/2G_{H2}$	-0.45 eV
**Intermediates**	**G-Mn SSCs**	**ΔG**	**Mn SSCs**
*NO	G_0 = -1182.63 eV		
*NOH	G_{1-1} = -1184.85 eV	$\Delta G_{0\rightarrow 1-1} = G_{1-1} - G_0 - 1/2G_{H2}$	1.18 eV
*NHO	G_{1-2} = -1185.36 eV	$\Delta G_{0\rightarrow 1-2} = G_{1-2} - G_0 - 1/2G_{H2}$	0.67 eV
*NHOH	G_{2-2} = -1188.29 eV	$\Delta G_{1-2\rightarrow 2-2} = G_{2-2} - G_{1-2} - 1/2G_{H2}$	0.47 eV
*NH_2O	G_{2-3} = -1189.00 eV	$\Delta G_{1-2\rightarrow 2-3} = G_{2-3} - G_{1-2} - 1/2G_{H2}$	-0.23 eV

Table S3. Calculated values for free energy, and free energy change of the over-reduction and C-N coupling when *CYC is adsorbed at the site close to single Fe site.

	$*NH_2OH$	$*NH_2 + *OH$	$*NH_2OH + *CYC$	$*C_6H_{13}NO_2$	$*CHO + *H_2O$
G	G_1 = -1192.22 eV	G_2 = -1291.48 eV	G_3 = -1286.69 eV	G_4 = -1286.64 eV	G_5 = -1287.23 eV
ΔG	0 eV	0.74 eV	0.37 eV	0.42 eV	-0.17 eV

Table S4. Calculated values for the initial state, transition state and final state during the migration of *CYC from the far site to the close site.

	IS1	TS1	FS1
G	G_{IS} = -1262.27 eV	G_{TS} = -1261.45 eV	G_{FS} = -1262.46 eV
$G - G_{IS}$	0 eV	0.82 eV	-0.19 eV

Table S5. Calculated values for the adsorption energy of CYC at the different sites over Fe SSCs.

Sites	G-Fe SSCs	$*G_{CYC}$	Intermediates: *CYC
layer	G_{layer} = -1167.33 eV	G_{CYC} = -94.84 eV	
A site	G_A = -1162.27 eV	$*G_{CYC\text{-}A} = G_A - G_{CYC} - G_{layer}$	-0.10 eV
B site	G_B = -1162.36 eV	$*G_{CYC\text{-}B} = G_B - G_{CYC} - G_{layer}$	-0.19 eV
C site	G_C = -1162.46 eV	$*G_{CYC\text{-}C} = G_C - G_{CYC} - G_{layer}$	-0.29 eV
D site	G_D = -1162.45 eV	$*G_{CYC\text{-}D} = G_D - G_{CYC} - G_{layer}$	-0.28 eV

Table S6. Calculated values for the adsorption energy of CYC at the far sites and close sites over Fe-D GSCs with different defects.

Size	G_{layer}	$G_{CYC\ (close)}$	$G_{CYC\ (far)}$	$*G_{CYC}$	$*G_{CYC\ (close)}$	$*G_{CYC\ (far)}$
No defect	-1167.33 eV	-1162.27 eV	-1162.36 eV	$*G_{CYC} = G - G_{CYC} - G_{layer}$	-0.10 eV	-0.19 eV
One atom	-1155.06 eV	-1250.28 eV	-1149.81 eV		-0.39 eV	0.09 eV
Two atoms	-1143.29 eV	-1238.44 eV	-1138.01 eV		-0.17 eV	0.12 eV
Three atoms	-1132.61 eV	-1228.16 eV	-1227.33 eV		-0.71 eV	0.12 eV
Four atoms	-1105.23 eV	-1200.87 eV	-1199.25 eV		-0.80 eV	0.82 eV
Five atoms	-1099.79 eV	-1195.55 eV	-1194.09 eV		-0.93 eV	0.54 eV
Six atoms	-1089.08 eV	-1184.67 eV	-1183.85 eV		-0.76 eV	0.06 eV

Table S7. Calculated values for free energy, and free energy change for NO_2RR over Fe-D GSCs.

Intermediates	G-Fe-D GSCs	ΔG	Fe-D GSCs
*NO	G_0 = -1122.30 eV		
*NOH	$G_{1\text{-}1}$ = -1125.30 eV	$\Delta G_{0\rightarrow1\text{-}1} = G_{1\text{-}1} - G_0 - 1/2G_{H2}$	0.11 eV
*NHO	$G_{1\text{-}2}$ = -1126.14 eV	$\Delta G_{0\rightarrow1\text{-}2} = G_{1\text{-}2} - G_0 - 1/2G_{H2}$	-0.44 eV
*NHOH	$G_{2\text{-}2}$ = -1129.97 eV	$\Delta G_{1\text{-}2\rightarrow2\text{-}2} = G_{2\text{-}2} - G_{1\text{-}2} - 1/2G_{H2}$	-0.42 eV
$*NH_2O$	$G_{2\text{-}3}$ = -1130.63 eV	$\Delta G_{1\text{-}2\rightarrow2\text{-}3} = G_{2\text{-}3} - G_{1\text{-}2} - 1/2G_{H2}$	-1.09 eV

Table S8. Calculated values for free energy, and free energy change of the over-reduction and C-N coupling on Fe-D_4 CP.

	*NH_2OH	*NH_2 + *OH	*NH_2OH + *CYC (far)	*NH_2OH + *CYC (near)	*$C_6H_{13}NO_2$	*CHO + H_2O
G	G_1 = -1134.1 eV	G_2 = -1134.07 eV	G_3 = -1229.53 eV	G_4 = -1228.52 eV	G_5 = -1229.57 eV	G_5 = -1215.57 eV
ΔG	0 eV	0.03 eV	-0.59 eV	0.42 eV	-0.64 eV	-0.77 eV

Table S9. The EXAFS data fitting results of Fe SSCs and Fe-D GSCs samples.

Sample	Shell	*CN*	R (Å)	σ^2 (Å^2)	△E_0 (eV)	R factor
Fe SSCs	Fe-O	0.85	1.879	0.00325	-7.218	1.83%
	Fe-N	3.70	1.998			
Fe-D GSCs	Fe-O	1.05	1.887	0.00174	-6.981	2.11%
	Fe-N	2.80	2.006			

CN, coordination number; R, bonding distance; σ^2, the Debye−Waller factor; ΔE_0, inner potential shift; S_0^2 was fixed to 0.80; e Fitting range: $3 \le k$ (/Å) ≤ 8 and $1.0 \le R$ (Å) ≤ 3.0.

Table S10. Calculated values for atom economy (AE), E factors and raw-material cost (k$ per ton CHO) in previous reports and this work.

	N source	C source	Additional Electrolyte	AE	E factors	Raw-material cost (k$ per ton CHO)
Reference 16	0.5 M KNO_3	0.1 M ketone	1 M KOH	33.3%	8.04	4.2
Reference 17	0.1 M KNO_3	0.025 CYC	0.5 M KPi	40%	5.93	3.5
Reference 18	1.8 M KNO_3	0.5 M CYC	0.5 M Na_2CO_3-$NaHCO_3$	43.48%	2.56	3.3
Reference 19	1.0 M KNO_3	0.1 M CYC	0.5 M K_2CO_3	18.18%	11	7.4
Reference 20	0.1 M $NaNO_2$	0.01 M CYC	0.5 M PBS	18.18%	58.93	4.2
This work	0.5 M $NaNO_2$	0.5 M CYC	0.5 M Na_2CO_3	91.3%	1.51	1.4
This work$_{COAP}$	0.5 M $NaNO_2$	0.5 M CYC	No	89.1%	0.09	1.4

Cathode reaction: $NO_2^- + C_6H_{10}O + 3H_2O - 4e^- = C_6H_{11}NO + 5OH^-$
$NO_3^- + C_6H_{10}O + 4H_2O - 6e^- = C_6H_{11}NO + 7OH^-$
Anode reaction: $4OH^- + 4e^- = 2H_2O + O_2$
Total reaction 1 (NO_2^-): $NO_2^- + C_6H_{10}O + H_2O = C_6H_{11}NO + OH^- + O_2$
Total reaction 2 (NO_3^-): $2NO_3^- + 2C_6H_{10}O + 2H_2O = 2C_6H_{11}NO + 2OH^- + 3O_2$

AE = 2× n (oximes) / (n (N source) + n (C source))
E factor = m (waste) / m (product) = (m (electrolyte) + m (NH_3) + m (generated alkaline))
E factor (this work) = (m (rest CYC) + m (NH_3)+ m (NaOH)) / m (CHO)
E factor (this work$_{COAP}$) = (m (rest CYC) + m (NH_3)) / (m (CHO) + m (NaOH))
Raw-materials cost (k$ per ton CHO) = m (CYC) × 1161 + m ($NaNO_2$) × 529.49 / m (KNO_3) × 711.83

The E factors are defined as ratio of kgs waste to kgs product, and atom economy are defined as ratio of mol of product to sum of mol of starting materials. According to the total reactions from NO_3^- or NO_2^-, NH_3 and alkaline (KOH or NaOH) are the by-products at the cathode. Considering the existence of additional electrolytes, the recycling of additional electrolytes largely increases the separation cost for the whole process. Therefore, the additional electrolytes, alkaline and NH_3 are considered waste. On the contrary, as to this oxime-alkali process, additional electrolyte is avoided, and the alkaline can be theoretically separated via the simple evaporative crystallization. The generated NaOH is considered as the product in this work.

REFERENCE

1. Kohn, W., Sham, L. J. Self-consistent equations including exchange and correlation effects. *Phys. Rev.* **140**, A1133-A1138 (1965).
2. Kresse, G., Furthmüller, J. Efficient iterative schemes for ab initio total-energy calculations using a plane-wave basis set. *Phys. Rev. B* **54**, 11169-11186 (1996).
3. Kresse, G., Hafner, J. Ab initio molecular dynamics for open-shell transition metals. *Phys. Rev. B* **48**, 13115-13118 (1993).
4. Blöchl, P. E. Projector augmented-wave method. *Phys. Rev. B* **50**, 17953-17979 (1994).
5. Grimme, S., Antony, J., Ehrlich, S., Krieg, H. A consistent and accurate ab initio parametrization of density functional dispersion correction (dft-d) for the 94 elements h-pu. *The Phys. Rev. B* **132**, 154104 (2010).
6. Grimme, S. Semiempirical gga-type density functional constructed with a long-range dispersion correction. *J. Comput. Chem.* **27**, 1787-1799 (2006).
